# The Synergistic Effect of Chemical Carcinogens Enhances Epstein-Barr Virus Reactivation and Tumor Progression of Nasopharyngeal Carcinoma Cells

**DOI:** 10.1371/journal.pone.0044810

**Published:** 2012-09-14

**Authors:** Chih-Yeu Fang, Sheng-Yen Huang, Chung-Chun Wu, Hui-Yu Hsu, Sheng-Ping Chou, Ching-Hwa Tsai, Yao Chang, Kenzo Takada, Jen-Yang Chen

**Affiliations:** 1 National Institute of Cancer Research, National Health Research Institutes, Zhunan Town, Miaoli County, Taiwan; 2 Graduate Program of Biotechnology in Medicine of National Tsing Hua University and National Health Research Institutes, Hsinchu, Taiwan; 3 Institute of Biotechnology, Department of Life Sciences, National Tsing Hua University, Hsinchu, Taiwan; 4 Department of Microbiology, College of Medicine, National Taiwan University, Taipei, Taiwan; 5 National Institute of Infectious Diseases and Vaccinology, National Health Research Institutes, Tainan, Taiwan; 6 Department of Tumor Virology, Institute for Genetic Medicine, Hokkaido University, Sapporo, Japan; Karolinska Institutet, Sweden

## Abstract

Seroepidemiological studies imply a correlation between Epstein-Barr virus (EBV) reactivation and the development of nasopharyngeal carcinoma (NPC). *N*-nitroso compounds, phorbols, and butyrates are chemicals found in food and herb samples collected from NPC high-risk areas. These chemicals have been reported to be risk factors contributing to the development of NPC, however, the underlying mechanism is not fully understood. We have demonstrated previously that low dose *N*-methyl-*N*’-nitro-*N*-nitrosoguanidine (MNNG, 0.1 µg/ml) had a synergistic effect with 12-*O*-tetradecanoylphorbol-13-acetate (TPA) and sodium butyrate (SB) in enhancing EBV reactivation and genome instability in NPC cells harboring EBV. Considering that residents in NPC high-risk areas may contact regularly with these chemical carcinogens, it is vital to elucidate the relation between chemicals and EBV and their contributions to the carcinogenesis of NPC. In this study, we constructed a cell culture model to show that genome instability, alterations of cancer hallmark gene expression, and tumorigenicity were increased after recurrent EBV reactivation in NPC cells following combined treatment of TPA/SB and MNNG. NPC cells latently infected with EBV, NA, and the corresponding EBV-negative cell, NPC-TW01, were periodically treated with MNNG, TPA/SB, or TPA/SB combined with MNNG. With chemically-induced recurrent reactivation of EBV, the degree of genome instability was significantly enhanced in NA cells treated with a combination of TPA/SB and MNNG than those treated individually. The Matrigel invasiveness, as well as the tumorigenicity in mouse, was also enhanced in NA cells after recurrent EBV reactivation. Expression profile analysis by microarray indicates that many carcinogenesis-related genes were altered after recurrent EBV reactivation, and several aberrations observed in cell lines correspond to alterations in NPC lesions. These results indicate that cooperation between chemical carcinogens can enhance the reactivation of EBV and, over recurrent reactivations, lead to alteration of cancer hallmark gene expression with resultant enhancement of tumorigenesis in NPC.

## Introduction

Nasopharyngeal carcinoma (NPC) is a common cancer in the southern part of China, Taiwan and southeastern Asia. Genetic, environmental and microbial factors have been incriminated in the carcinogenesis of NPC [1], [2]. Epstein-Barr virus (EBV), a human gamma-herpesvirus, is the etiological agent of infectious mononucleosis and is implicated in the development of several human malignancies, including NPC. Retrospective studies revealed that the sera of NPC patients contained antibodies against EBV prior to diagnosis and prospective studies also indicated that individuals with elevated antibodies against EBV have a higher risk of the development of NPC [3]–[5]. In addition, seroepidemiological studies revealed that populations living in NPC high risk areas have higher frequencies and levels of antibodies against EBV [6], [7]. These studies strongly support the notion that EBV plays an etiological role in the carcinogenesis of NPC.

The consumption of Cantonese-style salted fish has been associated with NPC since 1972 [8]. It was found that volatile *N*-nitrosamines and their precursors are present in foodstuffs from NPC high risk areas and are a potential etiological factor for NPC [9], [10]. Preserved food samples from NPC high risk areas were found to contain EBV inducers and mutagens, as well as *N*-nitrosamines [11]. Moreover, it has also been shown that various chemicals, including phorbol esters and *n*-butyrate which are present in several herbal medicines and food sources, can induce the EBV lytic cycle and may be involved in the tumorigenesis of NPC [12]–[14]. It has been shown that regions of China with a high annual incidence of NPC were colocalized with those where herbal drugs containing phorbol esters are commonly used [15]. These results suggest that chemical carcinogens may contribute to the carcinogenesis of NPC. However, the underlying mechanism has not been extensively studied yet [16].

Chromosomal abnormalities have been detected in NPC tissues [17]–[19]. Taking chromosomal 3p loss of heterozygosity (LOH) as a marker, NPC patients were reported to have the highest frequency of 3p deletion, followed by residents in NPC high risk areas, while the LOH frequency on 3p is low in areas with low incidence of NPC [20]. This observation suggested that genome instability is closely associated with the development of NPC. Given that both chemicals and the virus have been shown to be co-carcinogens in the development of cancer [21], it would be interesting to examine the interplay between EBV and chemical carcinogens and their effects on the genome instability of NPC.

Life-long infection with EBV is ubiquitous among human adults worldwide, yet a very high incidence of NPC is predominately in specific geographical regions [22]. Although human genetic variation has been linked to the development of NPC [23], it is apparent that differences in lifestyle and dietary factors, in concert with environmental factors, are likely to increase the risk of developing this cancer and lead to the unique distribution of NPC. EBV infection, chemical carcinogens and their interaction may play a substantial role in the development of NPC. Elevation of antibodies against EBV has been considered as a marker of EBV reactivation [22], [24], [25]. Antibody titers against EBV have been shown to increase with the stage of NPC [26], [27], decrease after therapy with remission [26], and increase prior to relapse and metastasis [26], [28]. These observations suggest that EBV reactivation is closely related to the development and relapse of NPC. We have shown previously that 12-*O*-tetradecanoylphorbol-13-acetate (TPA) and sodium butyrate (SB) can induce the reactivation of EBV to enhance the genome instability and tumorigenisity of NPC cells [29]. This result supports the notion that chemical reactivation of EBV may contribute to the carcinogenesis of NPC [15]. Our recent study showed that the reactivation of EBV could be initiated in NPC cells by *N*-methyl-*N*’-nitro-*N*-nitrosoguanidine (MNNG, a nitrosamide). Moreover, when treated in combination, low dose MNNG (0.1 µg/ml) had a synergistic effect with TPA/SB in enhancing EBV reactivation [30]. Because residents of areas with a high risk of NPC may come into contact with these carcinogens in a long-term, low dose, repeated manner, we sought to determine the consequence of repeated exposure of EBV-harboring nasopharyngeal cells to carcinogens. Additionally, although EBV reactivation has been shown to enhance genome instability, its impact on the alteration of cellular gene expression is unknown. Herein, we developed an in vitro NPC cell model system to simulate the repeated exposure to carcinogens by repeated treatment of NPC cells with a combination of TPA, SB and MNNG. This treatment induced recurrent reactivation of EBV in EBV-positive NPC cells and the degree of genome instability in these cells were enhanced significantly with combined TPA/SB and MNNG treatment, rather than when they were treated with the chemicals individually. The invasiveness, as well as tumorigenicity, was also enhanced in NPC cells after recurrent EBV reactivation. The genes with altered expression in EBV-positive NPC cells following recurrent reactivations were identified by expression profile analysis and can be categorized into many carcinogenesis-related clusters. Notably, several of these aberrations corresponded to alterations in clinical NPC lesions. Taken together, our study provides evidence for cooperation between chemical carcinogens and EBV reactivation in the enhancement of genome instability and alteration of cancer hallmark gene expression, resulting in a contribution to the carcinogenesis of NPC.

## Results

### MNNG Enhances TPA/SB-induced EBV Reactivation

**Figure 1:**
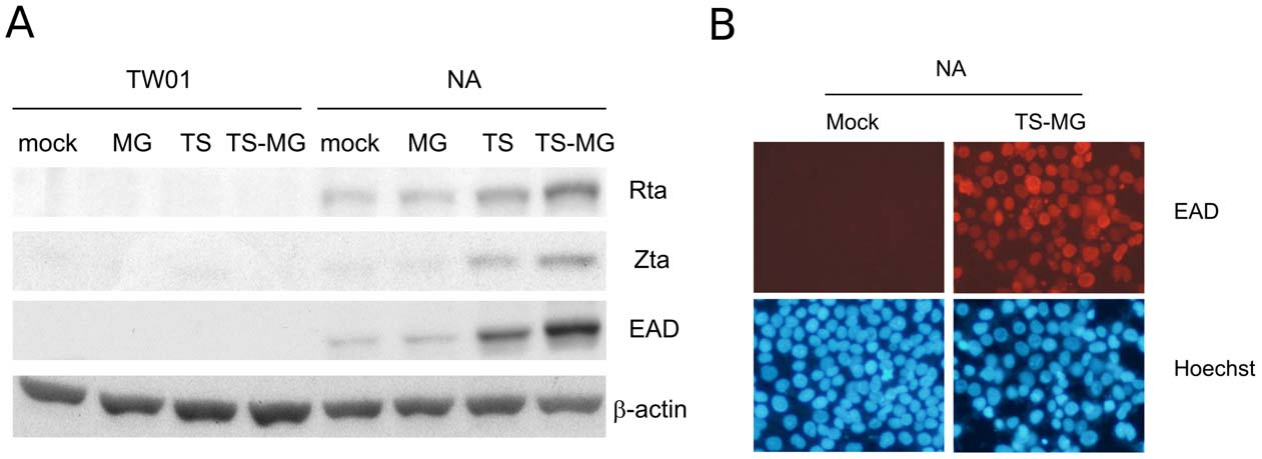
MNNG enhances TPA/SB-induced EBV reactivation. Cells were mock or treated with indicated chemicals. (A) Cell lysates were subjected to western blot analysis of EBV lytic protein Rta, Zta, and EAD. Reactivation of EBV was enhanced by co-treatment of MNNG and TPA/SB in NA cells, as determined by the expression level of lytic proteins. β-actin was used as a loading control. MG, TS and TS-MG indicate cells treated with MNNG, TPA/SB, and TPA/SB combined with MNNG, respectively. (B) Over 70% of NA cells were induced into EBV reactivation by the combination treatment of MNNG and TPA/SB, as determined by immunofluorescence assay of EAD. The nuclei of cells were stained with Hoechst 33258.

We have previously shown that MNNG can synergistically enhance TPA/SB-induced EBV reactivation [30]. A similar result is demonstrated in Figure 1. At mock-treated condition, a background level of EBV lytic markers was detected by immunoblot, which represents few spontaneous viral reactivation of EBV-positive NA cells under normal condition (Figure 1A). Treatment of 0.1 µg/ml MNNG did not induce discernible viral reactivation in NA cells, as compared to the mock-treated group. Treatment of TPA (10 ng/ml) and SB (1 mM) can induce EBV into reactivation, as demonstrated by the expression of EBV lytic gene BRLF1 (Rta), BZLF1 (Zta), and BMRF1 (EAD). Interestingly, although 0.1 µg/ml MNNG induced indiscernible EBV reactivation, its combination with TPA and SB did significantly enhance the TPA/SB-induced EBV reactivation (Figure 1A). Under this condition, more than 70% NA cells were induced into EBV lytic cycle, as determined by EAD immunofluorescence (Figure 1B). This result indicated that MNNG can act synergistically with TPA/SB in enhancing EBV reactivation in NPC cells.

### Enhanced Recurrent EBV Reactivation by Chemical Carcinogens Leads to Increased Genome Instability in NA Cells

**Figure 2:**
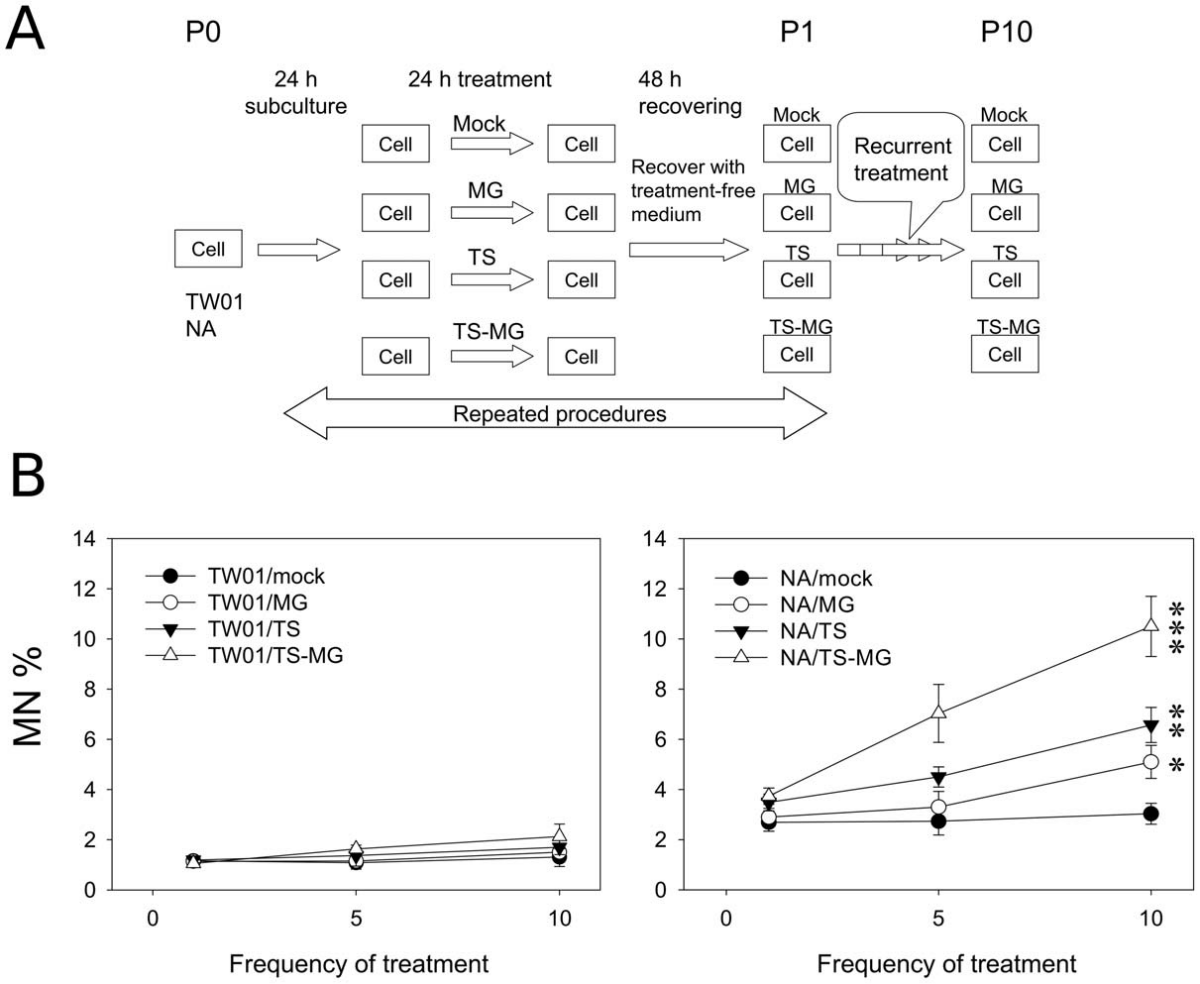
Recurrent EBV reactivation concomitant with progressive genome instability in NPC cells. (A) Representative illustration of recurrent chemical treatment in NPC cells. Cells were mock or treated repeatedly with indicated chemicals. The cells at the beginning of this experiment were defined as passage 0 (P0) cells. After seeding, the cells were subjected to a 24 h period of mock or chemical treatment, and then were recovered by replacing with fresh medium for a further 48 h. The resulting cells were defined as passage 1 and the procedure was repeated for ten times. “P*n*” represents treated NPC cells, where *n* stands for the passage number of the cells. MG, TS and TS-MG indicate cells treated with MNNG, TPA/SB, and TPA/SB combined with MNNG, respectively. (B) Cells were stained with Hoechst 33258 and MN was examined using a fluorescence microscope. Occurrence of MN formation (MN%) is presented here as the percentage of the number of micronuclei-presenting cells per 1,000 cells analyzed and plotted as a function of the frequency of chemical treatment. At least 1,000 cells were counted for each experiment. Data indicate mean value in MN% (triplicates ± SD). *: *p*<0.05, **: *p*<0.01, ***: *p*<0.001, when compared to NA-P10/mock cells.

Since low dose MNNG has a synergistic effect with TPA and SB in inducing EBV reactivation (Figure 1). We postulated that frequent contact with these chemicals could trigger the recurrent reactivation of EBV and lead to the enhanced malignancy of NPC. To test this hypothesis, we carried out chemically-induced recurrent reactivation of EBV in EBV-positive NA cells in vitro to assess the impact of recurrent EBV reactivation on genome instability of the cells. A culture system was established to carry out a longitudinal study by repeated incubation of NPC cells in medium containing inducing chemicals (Figure 2A, detail described in Materials and Methods). The EBV-negative NPC-TW01 (TW01, the parental cell of NA) and EBV-positive NA cells were both engaged in this study for comparison of the effects between chemicals treatment alone (in TW01 cells) and chemically-induced EBV reactivation (in NA cells). The formation of micronuclei (MN) was used to determine how genome instability was affected by recurrent EBV reactivation. As shown in Figure 2B, formation of MN was low in EBV-negative TW01 cells (1.0% to ∼2.0%) throughout the 10 passages, regardless of the types of chemical treatment. This indicated that at a relatively low dose, these chemicals/carcinogens only induced a subtle increase in genome instability in EBV-negative cells after repeated treatment. The formation of MN in the mock treated-NA cells remained at a low level (approximately 2.6%) and did not rise significantly with progressive passages (*p*>0.05). It seemed that the intrinsic genome instability in mock-treated TW01 and NA cells remained relatively stable through the 10 passages. A steady increase of MN formation was observed in MNNG and TPA/SB-treated NA cells (2.8% to 5.0% and 3.3% to 6.0%, respectively). Furthermore, a profound increase in the formation of MN was observed in NA cells treated with combined TPA/SB and MNNG (3.7% to 10.3%) in proportion to the progression of treatment. These results indicate that, not only can MNNG and TPA/SB alone increase genome instability in EBV-positive NA cells, but the combination treatment with TPA/SB and MNNG can induce almost twice the amount of genome instability in NPC cells after repeated treatment. Although TW01 and NA cells both received the same scheme of repeated chemical treatment, only the EBV-positive NA cells were induced into recurrent EBV reactivation by chemicals (Figure 1 and [30]). Therefore, this result suggests that chemically-induced recurrent EBV reactivation can lead to the accumulation of genome instability in host cells, and the aggravation is proportional to the degree of EBV reactivation.

### Enhanced Invasiveness is Concomitant with Increased Genome Instability in NPC Cells after Recurrent EBV Reactivation

**Figure 3:**
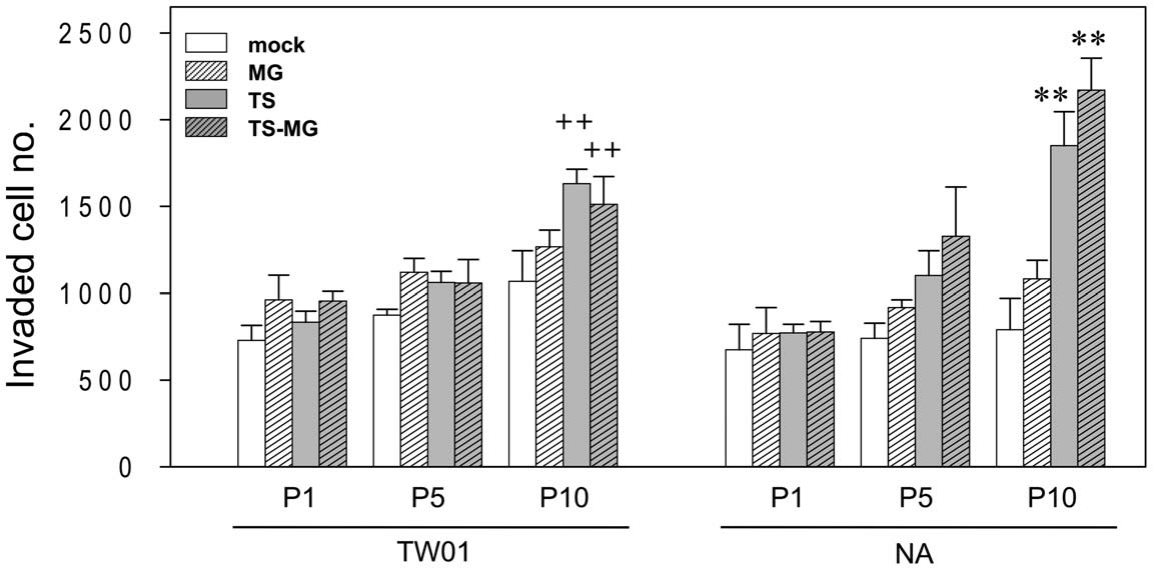
Recurrent EBV reactivations promote the invasiveness of NPC cells. EBV reactivation increases invasiveness, dependent on the number of rounds of reactivation. Mock or chemically-treated TW01 and NA cells were assayed for their ability to invade a Matrigel-coated membrane. Data indicate the average number of invading cells in three independent experiments (mean ± SD). ^++^: *p*<0.01, when compared to the TW01-P1/mock cells; **: *p*<0.01, when compared to the NA-P1/mock cells.

Because genome instability is a hallmark of cancer [31], the increase in the occurrence of MN observed in recurrently reactivated NA cells led us to question whether this chemically-induced recurrent EBV reactivation also enhance the carcinogenic phenotype of the NA cells. To examine this, an in vitro invasiveness assay was performed. As shown in Figure 3, for EBV-negative TW01 cells, a single treatment did not reveal marked differences in invasiveness. A slight increase in invasiveness was observed in chemically-treated cells at passage 5. When the treatments were extended to passage 10, the invasive ability was increased to about 2.0 fold for the TPA/SB and TPA/SB/MNNG-treated groups of TW01 cells (*p*<0.01; compared to TW01-P1/mock cells). This indicates that repeated treatment with chemical carcinogens, TPA/SB or MNNG, can increase the invasiveness of NPC cells, supposedly through the tumor-promoting properties of these compounds. For EBV-positive NA cells, a single treatment also did not reveal significant differences in the invasiveness of these cells. However, as the number of cycles of treatment increased, we observed a significant increase in invasiveness, with increasing passage number, under chemically-induced conditions. When comparing passage 10 to passage 1 of NA cells, the invading cells increased 1.6, 2.7, and 3.3 fold for the MNNG alone, TPA/SB alone and the combined TPA/SB and MNNG-treated groups, respectively (Figure 3). These results indicate that repeated treatment with low dose MNNG can increase slightly the invasive ability of NA cells. Furthermore, recurrent reactivations of EBV by TPA/SB treatment can markedly increase the invading cells, as we reported previously [29]. Notably, when the EBV reactivations were enhanced by combined treatment with TPA/SB and MNNG, a marked (3.3 fold) increase in invasiveness was observed. This indicates that enhanced EBV reactivation triggered by combined TPA/SB and MNNG not only can enhance the genome instability, but also result in a significant increase in the invasiveness of NPC cells. Taken together, these results indicate that chemically-induced recurrent EBV reactivations can enhance the invasiveness of NPC cells.

### Enhanced Recurrent EBV Reactivation by Chemical Carcinogens Aggravates the Tumor Progression of NPC Cells

**Figure 4:**
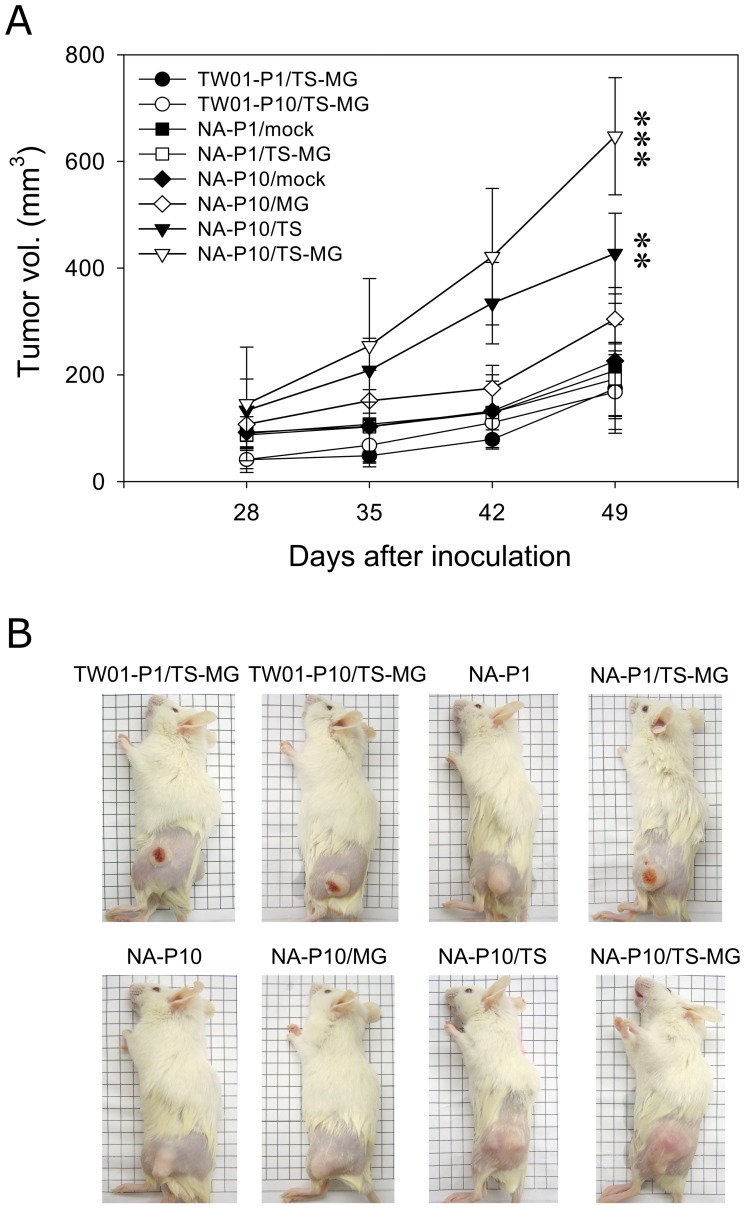
Recurrent EBV reactivations increase the tumorigenicity of NA cells *in vivo*. (A) The tumorigenicity of cells after recurrent EBV reactivation by chemical treatment was evaluated *in vivo* by injecting cells subcutaneously into SCID mice. The size of the tumors at the inoculation site was measured using a standard procedure at 7-day intervals post-injection. Data indicate mean tumor size (*n*≥8) ± SD. **: *p*<0.01, ***: *p*<0.001, when compared to NA-P10/mock cells. (B) Representative photographs of tumor nodules in the mice at day 52.

To evaluate the effect of chemically-induced recurrent EBV reactivations on tumor growth, a tumorigenicity assay was performed using SCID mice injected with variously treated NPC cells and monitored periodically by tumor volume. With only chemical treatment but no recurrent EBV reactivation, the tumor growth of TW01 cells exhibited no difference, regardless of one time or ten times of TPA/SB and MNNG treatment (TW01-P1/TS-MG and TW01-P10/TS-MG; Figure 4A and B). NA cells with one time of TPA/SB/MNNG-treatment (NA-P1/TS-MG) and ten times of mock treatment (NA-P10/mock) also did not reveal significant difference in tumor growth when compared with parental NA cells (NA-P1/mock). A slight increase in tumorigenicity was observed in mice inoculated with NA-P10 cells with repeated MNNG-treatment, albeit below statistical significance (NA-P10/MG; *p*>0.05, Figure 4A). A steady increase of tumor size was observed in mice bearing tumors from cells with recurrent TPA/SB and combined TPA/SB and MNNG treatment (NA-P10/TS and NA-P10/TS-MG, respectively). Dramatically increased tumor sizes were observed at day 49 in mice inoculated with NA-P10/TS-MG cells, compared to tumors obtained from mice inoculated with NA-P10/TS and NA-P10/MG, NA-P10/mock, NA-P1 and TW01 cells (Figure 4A). The inoculation sites photographed at day 52 also showed NA cells with accelerating tumorigenicity after recurrent EBV reactivation (Figure 4B). It seems that after 10 times of enhanced EBV reactivation, the NA-P10/TS-MG cells acquired the ability to propagate even faster than other cells in vivo. Taken together, these results show that recurrent EBV reactivation can enhance the tumorigenicity of NA cells and the aggravation is proportional to the degree and the frequency of EBV reactivation.

### Differentially Expressed Genes in NPC Cells after Recurrent EBV Reactivation

It is apparent that after chemically-induced recurrent EBV reactivation, the NA cells became more genetically unstable and gained enhanced malignancy. To determine which genes were altered and involved in the enhancement of malignant phenotypes during the progression, the expression profile of the NA-P1/mock and the NA-P10/TS-MG cells were analyzed by expression microarray. Under the criteria of *p*<0.01, >1.5-|fold change|, there were 618 probe sets expressed differentially, including 169 overexpressed and 449 underexpressed (result represented in Table S1). These genes were categorized by DAVID (the Database for Annotation, Visualization, and Integrated Discovery) to assign annotation groups, based on gene ontologies (GO). A complete list of the GO results can be found in Table S2. Pathways that are related to important cellular physiology are listed selectively in Table 1. Carcinogenesis-related clusters, including apoptosis, protease/peptidase, cell proliferation, response to extra/intra-cellular stimuli, and lipid/steroid metabolic processes were found to be altered (Table 1). Genes involved in five categories, including apoptosis, peptidase activity, cell proliferation, cell adhesion and antigen presentation, are presented in Table 2. Downregulation of apoptotic genes, such as DAPK2, HRK, TP53INP1, and upregulation of anti-apoptotic genes, including BCL11B, BAG1, SERPINB2, were noted. Genes involved in cell proliferation, including CCND2 and CDK6 were upregulated while CDKN1A, CDKN2B, IFITM1 and IGFBP7 were downregulated. Downregulation of many peptidases, including several kallikrein-related peptidases (KLK5, 6, 7, 8, and 10) and complement components (C1R, C1RL, C1S, and C2/CFB) also were observed. Repression of serine protease inhibitor SLPI and matrix metalloproteinases (MMP) inhibitor TIMP2 also were noted. MHC genes that are involved in antigen presentation, including HLA-B/-C/-F/-G, and genes responsible for antigen processing, CTSB and HSPA2, were underexpressed after recurrent EBV reactivation. The expression profiles of five of these genes were verified by quantitative RT-PCR (Figure S1). MIR17HG, a host gene for the micro RNA miR-17-92 cluster, was upregulated in NA-P10/TS-MG cells while HPGD (hydroxyprostaglandin dehydrogenase 15-(NAD)), FBXO32 (f-box only protein 32), TGM2 (transglutaminases 2) and LOXL4 (lysyl oxidase homolog 4) were downregulated. These results indicate that many genes differentially expressed in NPC cells after recurrent EBV reactivations are carcinogenesis-related genes.

**Table 1:** Selective presentation of 30 functional categories of the 618 probe sets by gene ontology.

Category	Genecount	*P*value
Response to organic substance	41	2.20E-05
Oxidation reduction	35	2.06E-04
Regulation of apoptosis*	32	0.039444
Peptidase activity*	31	0.001359
Regulation of cell proliferation*	30	0.072033
Response to wounding	27	0.003459
Lipid biosynthetic process	26	3.74E-06
Steroid metabolic process	25	1.86E-09
Negative regulation of gene expression	24	0.013345
Negative regulation of cellular biosyntheticprocess	23	0.063909
Serine-type peptidase activity	21	2.29E-07
Response to hormone stimulus	21	0.003223
Cholesterol metabolic process	17	4.09E-09
Response to extracellular stimulus	17	4.25E-04
Cofactor binding	17	0.002546
Negative regulation of apoptosis	17	0.037907
Non-coding RNA processing	13	0.005967
Cofactor metabolic process	13	0.008282
Anti-apoptosis	12	0.028939
Response to hypoxia	11	0.004124
Regeneration	9	6.18E-04
Regulation of inflammatory response	9	0.001179
Regulation of cell adhesion*	9	0.036925
Ribonucleotide metabolic process	9	0.052154
Peptidase inhibitor activity	9	0.077741
Glutathione metabolism	7	0.004067
Complement control module	7	0.005638
Metabolism of xenobiotics by cytochrome P450	7	0.009956
Antigen processing and presentation*	7	0.042248
Cyclin	6	0.010782

* Genes involved in the functional category are listed in table 2.

**Table 2:** List of genes involved in the indicated functional category from table 1.

Category	Genes involved in the functional category
Regulation of apoptosis	BCL11B, BCL6, BAG1, CD70, DFFA, DNAJB6, RAB27A, ANXA4, AEN, CAMK1D, CARD16, CTSB, CCL2, CDKN1A, DAPK2, HRK, HSPA1A/HSPA1B, HMGB1, ING3, IL7, MX1, NEFL, PCSK9, RYR2, SERPINB2, SOCS3, MUTED, TXNIP, TGM2, TNFAIP3, TP53INP1, ZC3H8
Peptidase activity	OMA1, SEC11C, CARD16, CTSB, C1R, C1RL, C1S, C2/CFB, DPP7, EML2, ERMP1, ECE2, KLK10, KLK5, KLK6, KLK7, KLK8, MMP28, PLAU, PCSK9, PRSS23, PRSS8, PSMB9, RHBDL1, SLPI, SCPEP1, SERPINB2, TMPRSS3, TPP1, TNFAIP3, ZMPSTE24
Regulation of cell proliferation	DHCR7, BCL11B, BCL6, DBP, KLF4, TIMP2, ANG, CCL2, CCND2, CDK6, CDKN1A, CDKN2B, CTH, FGFBP1, FXN, GJA1, ID2, IGFBP7, IFITM1, IL7, KRT5, KRT6A, MVD, OSMR, ODC1, PLAU, SKAP2, TXNIP, TGM2, TP53I11
Regulation of cell adhesion	BCL6, FXYD5, L1CAM, CHRD, CDK6, GSN, SAA1, TGFBI, TGM2
Antigen processing and presentation	CTSB, HSPA1A/HSPA1B, HSPA2, KLRC1, HLA-B, HLA-C, HLA-F, HLA-G

### Several Differentially Expressed Genes in NPC Cells after Recurrent EBV Reactivation Corresponded to Alterations Observed in NPC Biopsies

**Figure 5:**
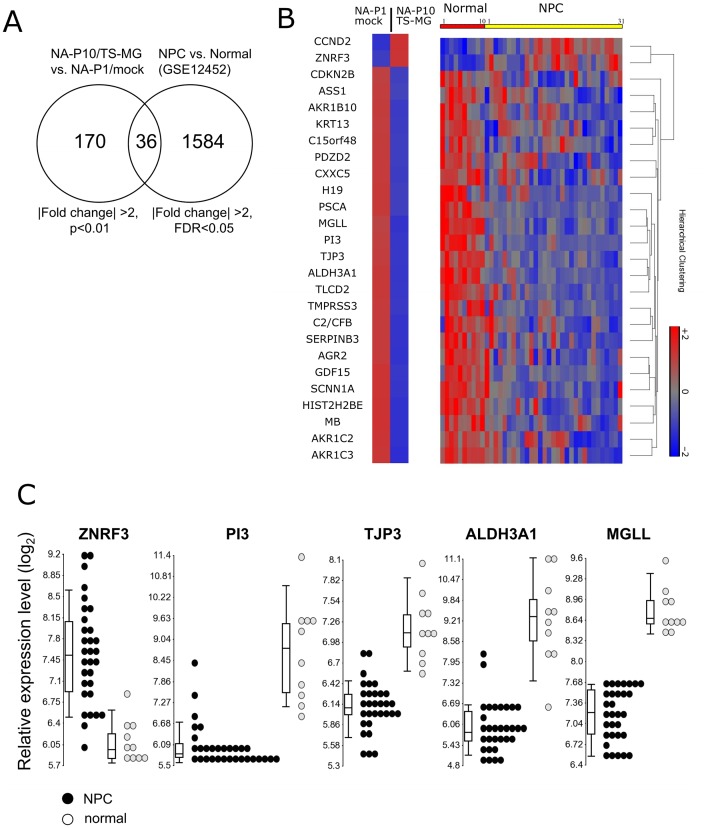
Differentially expressed genes in NPC cells after recurrent EBV reactivation corresponded to the alterations observed in NPC biopsies. (A) Differentially expressed probe sets of NA-P10/TS-MG and NA-P1/mock, and 31 NPC and 10 normal biopsies (GSE12452). The intersection between two data sets yielded a 36 common probe set which was presented in both groups. (B) Expression profile of 28 genes (derived from the 36 common probes) that were differentially expressed in NPC cell lines and biopsies. Hierarchical clustering was performed by shifting genes to mean of zero and scale to standard deviation of one. (C) Expression profile of five carcinogenesis-related genes in 31 NPC and 10 normal biopsies. Each circle represents the expression level of an individual biopsy. Boxplot indicates the median (central band) and the 10th and 90th percentile (lower and upper whiskers, respectively) of expression level. ZNRF3: zinc and ring finger 3; PI3: peptidase inhibitor 3, skin-derived; TJP3: tight junction protein 3; ALDH3A1: aldehyde dehydrogenase 3 family, member A1; MGLL: monoglyceride lipase.

Recurrent EBV reactivation altered the expression profile of NPC cells in vitro. Since many of these altered genes were carcinogenesis-related, we sought to determine whether these alterations corresponded to those observed in NPC lesions. To achieve this, a series of data sets containing the expression profiles of 31 laser captured, microdissected NPC biopsies and 10 normal nasopharyngeal (NP) tissue specimens (GSE12452) [32], [33], were included for comparison with the expression profiles of NPC cell lines in this study. The expression profiles of the NA-P1/mock and the NA-P10/TS-MG cells were analyzed here with the criteria of *p*<0.01, >2-|fold change|, which yielded a list of 170 differentially expressed probe sets (result represented in Table S3). Gene lists were created from GSE12452 (NPC vs. normal) using a cutoff of false discovery rate (FDR) <0.05, >2-|fold change|, which yielded a list of 1584 differentially expressed probe sets. Intersection between two probe sets yields 36 probes that were commonly differentially expressed between the groups (Figure 5A). Figure 5B illustrates the expression profile of 26 genes (derived from the 36 probe sets) of NPC cell lines and normal NP and NPC specimens. Upregulation of CCND2 and ZNRF3 was observed in NA-P10/TS-MG cells and in NPC biopsies, while other genes, including ALDH3A1, ASS1, MGLL, PSCA, and TJP3, were downregulated as compared to NA-P1/mock cells and normal NP tissues, respectively. Expression profiles of five genes in biopsies, including ZNRF3, PI3, TJP3, ALDH3A1, and MGLL are shown in Figure 5C. Several genes corresponding to the 170 probes in NA-P10/TS-MG cells are categorized as carcinogenesis-related oncogenes and tumor suppressor genes (Table S4). Literatures from previous studies of other malignancies, indicating those genes as putative oncogenes/tumor suppressor genes, are indexed in this table (listed references in Document S1). The alterations corresponding to the 26 genes of the 31 NPC biopsies are also noted. In this table, six genes were found to be overexpressed while 58 genes were underexpressed (Table S4) in NA-P10/TS-MG cells after recurrent EBV reactivation. Alterations of these oncogenes and tumor suppressor genes were reported previously in many types of cancers. Interestingly, there are ten genes, which were upregulated in many, if not all, cancers and have been considered as putative oncogenes, and these were downregulated in NA-P10/TS-MG cells (Table S4C). Among these genes, the downregulation of MGLL, ALDH3A1, SERPINB3, PSCA and H19 also were observed in NPC lesions (Table S4C). Taken together, these results indicate that genes differentially expressed in NPC cells after recurrent EBV reactivation are carcinogenesis-enhancing and correspond to the alterations observed in NPC biopsies.

## Discussion

Many dietary ingredients, particularly *N*-nitroso compounds, have been suggested to be associated with the development of NPC [9], [34]. Epidemiological studies show that the ingestion of Cantonese-style salted fish is a causative factor for NPC in Southern China [8]. Volatile nitrosamines are known to be present in foods from NPC high risk areas and considered to be the etiological factors for NPC [9], [10]. The total volatile nitrosamines were estimated to be in the range of 0.028 to 4.54 mg/kg in Chinese salted fish [10]. Under this condition, the exposure of volatile nitrosamines may reach significant level for people who consume them regularly. Interestingly, the highest incidence of NPC was observed among the boat people of Hong Kong who consumed salted fish as a major food source during weaning age [8]. Another common route for contact with *N*-nitroso compounds is tobacco products, which contain various types of nitrosamines that have been proved to be highly carcinogenic [35]. A recent report indicates that the hazard ratio of developing NPC is 3.0 for ≥30 pack-year of cumulative cigarette smoking persons when compared with <30 pack-year as the reference in Taiwan [36]. A study of Thai cigarettes indicated that the exposure level for mainstream smoker was 20.3 to 100.4 ng/cigarette for volatile nitrosamine, while total tobacco-specific nitrosamine was 88 to 1,580 ng/cigarette [37]. These reports suggest that the exposure level of nitrosamines for cigarette smokers, especially those with heavier habitat (>1 pack/20 cigarettes per day), may be quite substantial, considering that the nasopharyngeal tissue is one of the primary regions that contacts with these carcinogens. In addition to *N*-nitroso compounds, TPA and SB were also reported to induce the EBV lytic cycle efficiently [12], [13]. The choice of TPA and SB for EBV reactivation is a reflection of previous studies indicating that regions with a high annual incidence of NPC were colocalized with those where herbal drugs containing phorbol esters are consumed [15], [38]. Croton oil, which contains up to 1.6% of phorbols [39], is used commonly in traditional medicine in Southern China and Sri Lanka. The observation that the use of croton oil is overlapped with regions with high incidence of NPC have prompted scientists to propose that phorbol esters is involved in NPC carcinogenesis [15], [38]. Additionally, butyrates are fatty acids found in dairy products, especially in butter. When butter goes rancid, butyric acid is liberated from the glyceride by hydrolysis. Microorganisms in oral cavity and digestive tracks also produce butyrates as the end product of anaerobic fermentation. Consumption in certain North African regions of rancid butter or rancid sheep fat, which contains 3 to 4% of butyrates, also had been linked to NPC development [40]. Taken together, these studies reflect that frequent consumption of salted fish and tobacco products, croton oil or rancid butters, which contain nitrosamines, phorbols and butyrates respectively, can increase the incidence of NPC. However, the underlying mechanisms have not been elucidated. In our study, we demonstrated that these chemicals can induce or enhance EBV reactivation, and upon repeated exposure, lead to genome instability of host cell and increase its carcinogenicity.

Although studies have indicated that *N*-nitroso compounds, phorbols, and butyrates are the etiological cofactors of NPC, it is not known if these chemicals act simultaneously in vivo. Epidemiological investigation for the combination effects of any two of these factors is unavailable. However, since salted fish and rancid butter consumption, cigarette smoking, and herbal medicine are common in these etiological regions, it is possible that these chemicals may act synergistically in inducing EBV reactivation. Altogether, our study provides a possible mechanism to elucidate how these EBV inducers can contribute to the carcinogenesis of NPC. Even if the contact dosage is low, through regular and repeated exposure to single or more of these chemicals, some of the EBV-positive nasopharyngeal cells can be induced into recurrent reactivation and lead to accumulation of genome instability. Via selection, the reactivated cells with enhanced malignancy may emerge.

For EBV-positive NA cell and its parental, EBV-negative TW01 cell, the result after repeated chemical treatment is dramatically different. In this study, the dosage of treatments, either single or combined, did not induce profound increase of genome instability in TW01 cells (Figure 2B). However, after 10 times of treatment, the invasiveness of TW01 cells did increase to two-fold (Figure 3). This might reflect the intrinsic properties of these chemicals as tumor-promoting agents. TPA, an activator of the protein kinase C, is known to be involved in cell proliferation, differentiation, and migration [41]. Prolonged treatment with TPA had been shown to increase migration and invasion in tumor cells [42]. SB, a histone deacetylase inhibitor, is known to inhibit proliferation, induce apoptosis and differentiation in cancer cells [43]. MNNG is an alkylating agent, which induces DNA strand breaks and is involved in the carcinogenesis of model animals [44]. When treated in combination at low dosage, these chemicals had moderate effect on genome instability of TW01 cells throughout the ten passages (Figure 2B). In contrast, treatment of these chemicals induced EBV reactivation in NA cells (Figure 1). Accumulation of genome instability was observed as the frequency of treatment increased, while concomitant with marked elevation in invasiveness and tumorigenesis (Figure 2, 3 and 4). Epidemiological studies have suggested that contact with chemical carcinogens can contribute to the carcinogenesis of NPC, possibly through its tumor-promoting properties. Latent infection of EBV and expression of latent genes in EBV-infected cells were also shown to promote NPC progression [45]. However, since these chemicals can trigger the EBV into reactivation, a profound increase in carcinogenesis was observed in this study. Therefore, we proposed that, although chemical carcinogens and latent EBV infection do contribute independently to the carcinogenesis of NPC, recurrent EBV reactivation induced by chemicals may make a much significant contribution. Together, the cooperation of these three factors (chemical carcinogens, latent EBV infection and chemically-induced EBV reactivation) may lead to the dramatic change of genome instability and consequently contribute to the carcinogenesis of NPC.

In this study, recurrent EBV reactivation is markedly associated with accumulation of genome instability (Figure 2B). This result indicates that induction of the EBV lytic cycle can contribute to the damage of host cell genomes. In our previous study, EBV DNase, uracil DNA-glycosylase and major DNA-binding protein were found to increase MN formation and DNA damage when expressed in NPC cells [29]. Among these genes, a striking effect was found to be contributed by the EBV DNase, an early lytic gene in EBV replication [29]. In addition to induction of DNA damage, our previous study revealed that EBV DNase can repress DNA repair and increase genetic mutations [46]. We have also found that EBV BHRF1, a homologue of the BCL-2 protooncogene, and BALF3, an EBV DNA terminase, can increase MN formation when expressed in epithelial cells (unpublished observations). We have also shown that EBV BGLF4 induces premature chromosome condensation [47], which can contribute to genome instability of the host cell [48]. The expression of EBV DNase has been detected in NPC tissues [49], as have other EBV lytic genes such as Zta, Rta and gp220 [50]–[52]. Reactivation and replication of EBV has been reported in the nasopharyngeal region, indicating that EBV reactivation may not be an uncommon event in vivo [32], [53]. Conversely, inhibition of EBV reactivation by interfering RNA specific to the EBV immediate early gene Zta could abolish MN formation. We have shown that, even in the presence of EBV-inducing carcinogens, blocking EBV reactivation by siZta led to reduce genome instability in NPC cells [30]. These studies indicate that, in addition to the well-accepted concept of contribution to carcinogenesis of NPC by latent EBV infection, expression of lytic genes during EBV reactivation can induce DNA damage, enhance genome instability and subsequently contribute to the carcinogenesis of NPC. Still, further studies are required to elucidate the underlying mechanisms of this viral contribution to host cell genome instability.

Following recurrent EBV reactivation, the invasiveness and tumorigenesis of NA cells were found to increase significantly (Figure 3 and 4). Previously, we have shown that recurrent EBV reactivation can lead to chromosomal structural aberrations and alterations of DNA copy-numbers [29]. Hence, in this study, we turned to explore the impact of recurrent EBV reactivation on the gene expression of host cells. The transcription profile assayed between the parental NA-P1/mock and the most aberrant NA-P10/TS-MG cells revealed that, after recurrent EBV reactivation, the expression of many genes was altered, including genes involved in various carcinogenic pathways (Table 1 and 2). Upregulation of anti-apoptotic genes, including BAG1, BCL11B and SERPINB2, and downregulation of apoptotic genes, such as DAPK2, HRK and TP53INP1, can promote tumor cell survival. A high frequency of promoter hypermethylation of DAKP2 in NPC patients has been reported [54]. Upregulation of CCND2 (cyclin D2) and CDK6 (cyclin-dependent kinase 6), which are involved in cell proliferation, and downregulation of CCNG2 (cyclin G2), CDKN1A, CDKN2B, IFITM1 and IGFBP7, have been noted after recurrent EBV reactivation. Upregulation of cyclin D2 and CDK6 has been observed in cancers [55], [56]. Conversely, the expression of the cyclin-dependent kinase inhibitors, CDKN2B and CDKN1A, were suppressed. The aberrant expression of these genes may promote NPC cell proliferation while escaping tight cell cycle control. Interestingly, alteration of these genes, including apoptotic and cell cycle-related (Table 2), may play a substantial role in the enhancement of tumorigenesis of NA-P10/TS-MG cells observed in SCID mouse assay (Figure 4). Moreover, repression of the MMP inhibitor TIMP2 and serine protease inhibitor SLPI can enhance tumor invasion and has been reported in several cancers [57], [58]. Downregulation of MHC genes that are involved in antigen presentation, including HLA-B/-C/-F/-G, and genes responsible for antigen processing, CTSB and HSPA2, may facilitating tumor cell evasion of immunosurveillance and, hence, increase tumor cell survival. Interestingly, deregulation of MHC genes has also been reported previously in the study of NPC biopsies with GSE12452 data sets [32].

List of putative oncogenes/tumor suppressor genes derived from the 170 differentially expressed probe sets is demonstrated (Table S4). Several of these aberrations correspond to alterations observed in NPC lesions (Figure 5 and Table S4). Interestingly, there are more deletions than amplifications of genes observed after 10 repeated EBV reactivations. It seems that reactivation of EBV has a tendency to suppress rather than promote host gene expression. Another interesting result also revealed in Table S4C is that several genes found to be overexpressed in many cancers were downregulated in NPC cell lines and biopsies. MGLL, a monoglyceride lipase overexpressed in several cancers [59], [60], was significantly downregulated in recurrent EBV reactivated cells and in all 31 NPC biopsies (Table S4C and Figure 5C). ALDH3A1, an aldehyde dehydrogenase involved in the acetaldehyde detoxification and steroids metabolism, had been shown to be upregulated in tumors and can promote cell proliferation [61], [62]. PSCA, a prostate stem cell antigen, has been reported to be upregulated in bladder, prostate and pancreatic cancers [63], [64] but is downregulated in esophageal and gastric cancers [65], [66]. PSCA seems to be a “Jekyll and Hyde” molecule that plays various roles, promoting or suppressing tumors depending on the cellular context [67]. The downregulation of these “putative oncogenes” in NPC cell lines and biopsies may indicate a unique feature of NPC. Nevertheless, how these discrepancies contribute to malignancy of NPC requires further investigation. The genes from the 170 probe sets were categorized according to the criteria of ten hallmarks of cancers proposed by Hanahan and Weinberg (Table S5) [31]. Except for one group, “Enabling Replicative Immortality”, which is an early event required for cell transformation, these genes can be sorted into the remaining nine groups, with “Invasion and Metastasis” and “Resisting Cell Death” being the most remarkable. These results reflect the fact that many genes expressed aberrantly after recurrent EBV reactivation promotes carcinogenesis. Based on these results, we proposed that the genomic alterations resulting from the recurrent EBV reactivation may serve to increase the mutability of the host genome, thereby accelerating the acquisition of alterations of cancer hallmark genes and promoting the carcinogenesis of NPC.

## Materials and Methods

### Ethical Statement

The mouse experiment of this study was carried out in strict accordance with the recommendations in the Guide for the Care and Use of Laboratory Animals of the National Institutes of Health. The protocol was approved by the Institutional Animal Care and Use Committee of National Health Research Institutes, Taiwan (NHRI-IACUC-098089-A). All efforts were made to minimize any possible suffering of the animals.

### Cell Lines

NPC-TW01 (TW01) is an EBV-negative nasopharyngeal carcinoma cell line derived from the nasopharyngeal tumors of a Chinese patient [68]. NA cell is an EBV-positive NPC cell derived from NPC-TW01 [69]. TW01 and NA cells were cultured in Dulbecco's modified Eagle medium (DMEM) supplemented with 10% fetal bovine serum (HyClone, Waltham, MA) at 37°C with 5% CO_2_. G418 (400 µg/ml, Ameresco, Solon, OH) was added to the medium of NA cells to maintain the EBV genome in the cells [69].

### Repeated Induction of EBV Reactivation

The EBV-negative TW01 cell and EBV-positive NA cell were both engaged in this study to compare the effects between chemicals treatment alone (in TW01 cells) and chemically-induced EBV reactivation (in NA cells). Repeated chemical exposures were achieved by incubating these cells periodically with (1) 0.1 µg/ml MNNG, or (2) TPA/SB (10 ng/ml and 1.0 mM), or (3) TPA/SB in combination with MNNG. A group of mock treated cells was included as negative controls. The cells at the beginning of this experiment were defined as passage 0 (P0) cells. 24 h after seeding, the cells were subjected to a 24 h period of mock or chemical treatment. After this 24 hr treatment, the cells were recovered by replacing the medium with fresh (treatment-free) medium and incubation for a further 48 h. The resulting cells were defined as passage 1 (P1, illustrated in Figure 2A). Cells from P1 were trypsinized and transferred and the process repeated again to obtain the P2 cells. “P*n*” represents treated NPC cells, where *n* stands for the passage number of the cells. A total of ten treatments were applied to the cells in this study. For convenience, MG, TS and TS-MG are used for description of cells treated with MNNG, TPA/SB, and TPA/SB combined with MNNG, respectively.

### Western Blot and Immunofluorescence Assay

The procedure for western blot assay of EBV lytic gene Rta, Zta and EAD, and immmunofluorescence assay of EAD in TPA/SB and MNNG treated NPC cells, are followed as describe previously [29].

### Analysis of Genome Instability by Micronucleus Formation

To determine whether the genome instability is a result of EBV reactivation in EBV-positive NPC cells following chemical treatment, we evaluated the occurrence of micronuclei (MN), which arise from acentric chromatids and have been recognized as a marker of genome instability [70]. The fixed cells were stained with Hoechst 33258 (0.2 µg/ml) (Sigma-Aldrich, St. Louis, MO). Micronuclei were examined using a fluorescence microscope and at least 1,000 cells were counted for each experiment.

### In vitro Invasiveness Assay

In vitro invasion assays were performed as described previously using HTS FluoroBlok inserts (Falcon, Cambridge, MA) [29]. The transwell membranes were first coated with Matrigel (Becton Dickinson, Franklin Lakes, NJ). 1×10^5^ cells were seeded onto the Matrigel-coated membranes and the inserts were incubated in 24-well plate for 48 h. After incubation, the membranes were fixed with methanol and stained with 50 µg/ml propidium iodide (Sigma-Aldrich). The cells that had invaded through the Matrigel and transmigrated to the lower surface of the polycarbonate membrane were photographed under a fluorescence microscope and the cell number was calculated using AIS software (Imagine Research Inc., Ontario, Canada).

### In vivo Tumorigenesis Assay

Six-week-old severe combined immunodeficiency (SCID) mice were used for this study. Mice were kept under sterile conditions. Tested cells (2×10^6^ cells in each case) were resuspended in serum-free DMEM and injected subcutaneously into the dorsal flanks of SCID mice. Injected mice were examined weekly and tumor volumes were estimated from their length (*l*) and width (*w*), as measured by calipers, using the formula tumor volume* = l w* ^2^×0.52.

### RNA Extraction and Semi-quantitative Real-time PCR

RNA was extracted from NPC cells using the Qiagen RNeasy mini kit (Qiagen, Valencia, CA). The quality of total RNA samples was examined by Bioanalyzer 2100 (Agilent, Santa Clara, CA) to ensure the integrity of RNA samples (RNA integrity numbers >8). For quantification of genes of interest, the RNA samples were reverse transcribed into cDNA using the QuantiTect Reverse Transcription Kit (Qiagen). One twentieth of the cDNA was used in semi-quantitative real-time PCR (qRT-PCR) for genes of interest using QuantiTect SYBR Green PCR kit (Qiagen). The calculations for determining the level of gene expression were made using the cycle threshold (Ct) method. Utilizing the human GAPDH gene as the internal control, the relative quantitation values of a target template for each sample were expressed as 2^−ΔΔCt^, where ΔΔCt = ΔCt ^NA-P10/TS-MG^ −ΔCt ^NA-P1/mock^. The primer sequences used in this study are listed in Table S6.

### Gene Expression Profiling

The expression profile of the NA-P1/mock and the NA-P10/TS-MG cells were analyzed by expression microarray. The analyses used whole-genome Affymetrix U133 Plus 2.0 human oligonucleotide microarrays containing over 47,000 transcripts and variants including 38,500 well-characterized human genes. Preparation of cRNA, hybridizations, washes, and detection were carried out according to the protocol recommended by the supplier (http://www.affymetrix.com/index.affx). A duplicate of each sample was analyzed and the result data set was deposited in the GEO database (http://www.ncbi.nlm.nih.gov/geo/) with accession number GSE38453.

### Public Domain Data

A data set of expression profile of NPC, GSE12452, which was deposited by Paul Ahlquist in GEO [32], [33], was included in the following data analysis. This data set includes expression profiles of 31 laser captured, microdissected NPC biopsies and 10 normal healthy nasopharyngeal tissue specimens, also assayed by Affymetrix U133 Plus 2.0 microarrays.

### Gene Expression Data Analysis

Gene expression data were imported into the Partek Genomics Suite 6.5 (Partek, St Louis, Mo) using default parameters. Raw data were preprocessed by background correction, normalization, and summarization using robust multiarray average analysis, and the expression data were log_2_ transformed. Differential expression analysis was performed by 1-way analysis of variance (ANOVA). Differentially expressed gene lists from NPC cell lines (NA-P10/TS-MG vs. NA-P1/mock) were first created using a cutoff of *p*<0.01, >1.5-|fold change|. To identify genes that corresponded to alterations observed in NPC lesions, the gene list of NPC cell lines (NA-P10/TS-MG vs. NA-P1/mock) were created using a cutoff of *p* <0.01, >2-|fold change|. Gene lists from GSE12452 (NPC vs. normal) were created using a cutoff of false discovery rate (FDR) <0.05, >2-|fold change|. The resulting gene lists from the NPC cell lines and the GSE12452 data set were compared further by probe set intersection. Genes that appeared in both lists were identified accordingly. Hierarchical clustering was performed by shifting gene expression to mean of zero and scale to standard deviation of one in the Partek Genomics Suite.

### Gene Ontology/pathway Analysis

The Database for Annotation, Visualization, and Integrated Discovery (DAVID) Bioinformatics Resource 6.7 from the National Institute of Allergy and Infectious Diseases (NIAID/NIH; http://david.abcc.ncifcrf.gov/) was applied to generate clusters of functionally related genes. The Functional Annotation Clustering tool was used to generate clusters of overrepresented Gene Ontology (GO) terms [71], [72]. For categorizing genes according to the criteria of ten hallmarks of cancers [31], the QuickGO of the Gene Ontology Annotation (UniProt-GOA, http://www.ebi.ac.uk/QuickGO/) Database was applied to determine the function and process of examined genes [73].

### Statistical Analysis

Differences between multiple groups were analyzed by one-way ANOVA with Tukey’s method for pairwise comparisons. *T*-test was used for comparisons of two groups. All statistical tests were two sided, and *p*<0.05 was considered to be statistically significant.

## Supporting Information

Figure S1 **Quantitative RT-PCR validation of genes that were differentially displayed in NA-P1/mock and NA-P10/TS-MG cells.** The expression level of gene in NA-P1/mock cells was adjusted as the base line (1-fold) and the relative expression level of gene in NA-P10/TS-MG cells was determined accordingly. Data indicate the mean expression level ± SD. MIR17HG: miR-17-92 cluster host gene; HPGD: hydroxyprostaglandin dehydrogenase 15-(NAD); FBXO32: f-box only protein 32; TGM2: transglutaminases 2; LOXL4: lysyl oxidase homolog 4.
(PDF)

Table S1 **List of genes that were altered after recurrent EBV reactivation.** To determine which gene expression levels were altered during recurrent EBV reactivation, the expression profile of the NA-P1/mock and the NA-P10/TS-MG cells were analyzed by expression microarray. Under the criteria of *p*<0.01, >1.5-|fold change|, there were 618 probe sets expressed differentially, including 169 overexpressed and 449 underexpressed.
(XLS)

Table S2 **Ontology categorization of genes that were altered after recurrent EBV reactivation.** The differentially expressed 618 probe sets were categorized by DAVID (the Database for Annotation, Visualization, and Integrated Discovery) to assign annotation groups, based on gene ontologies (GO).
(XLS)

Table S3 **List of genes that were altered after recurrent EBV reactivation with fold change >2.** The expression profiles of the NA-P1/mock and the NA-P10/TS-MG cells were analyzed with the criteria of *p*<0.01, >2-|fold change|, which yielded a list of 170 differentially expressed probe sets.
(XLS)

Table S4 **Putative oncogenes and tumor suppressor genes that were altered after recurrent EBV reactivation.** Several genes corresponding to the 170 probes in NA-P10/TS-MG cells are categorized as carcinogenesis-related oncogenes and tumor suppressor genes. Literatures from previous studies of other malignancies, indicating those genes as putative oncogenes/tumor suppressor genes, are indexed in this list. The alterations corresponded to the 26 genes of the 31 NPC biopsies are also noted.
(PDF)

Table S5 **Differentially displayed genes in NA-P10/TS-MG cells arranged by the criteria of ten hallmarks of cancers.** The genes from the 170 probe sets were categorized according to the criteria of ten hallmarks of cancers proposed by Hanahan and Weinberg [31]. Genes were categorized by their function and involved process provide by gene ontologies.
(PDF)

Table S6 **Primers used in semi-quantitative real-time PCR for genes of interest.**
(PDF)

Document S1 **List of cited references in Table S4 and S5.**
(PDF)
