# Supplementary material for: The Synergistic Effect of Chemical Carcinogens Enhances Epstein-Barr Virus Reactivation and Tumor Progression of Nasopharyngeal Carcinoma Cells
Source: PLoS One. 2012 Sep 14;7(9):e44810. doi: 10.1371/journal.pone.0044810 (PMC3443098; doi:10.1371/journal.pone.0044810)
Supplement: Figure S1 — Quantitative RT-PCR validation of genes that were differentially displayed in NA-P1/mock and NA-P10/TS-MG cells. The expression level of gene in NA-P1/mock cells was adjusted as the base line (1-fold) and the relative expression level of gene in NA-P10/TS-MG cells was determined accordingly. Data indicate the mean expression level ± SD. MIR17HG: miR-17-92 cluster host gene; HPGD: hydroxyprostaglandin dehydrogenase 15-(NAD); FBXO32: f-box only protein 32; TGM2: transglutaminases 2; LOXL4: lysyl oxidase homolog 4. (PDF) [file pone.0044810.s001.pdf]

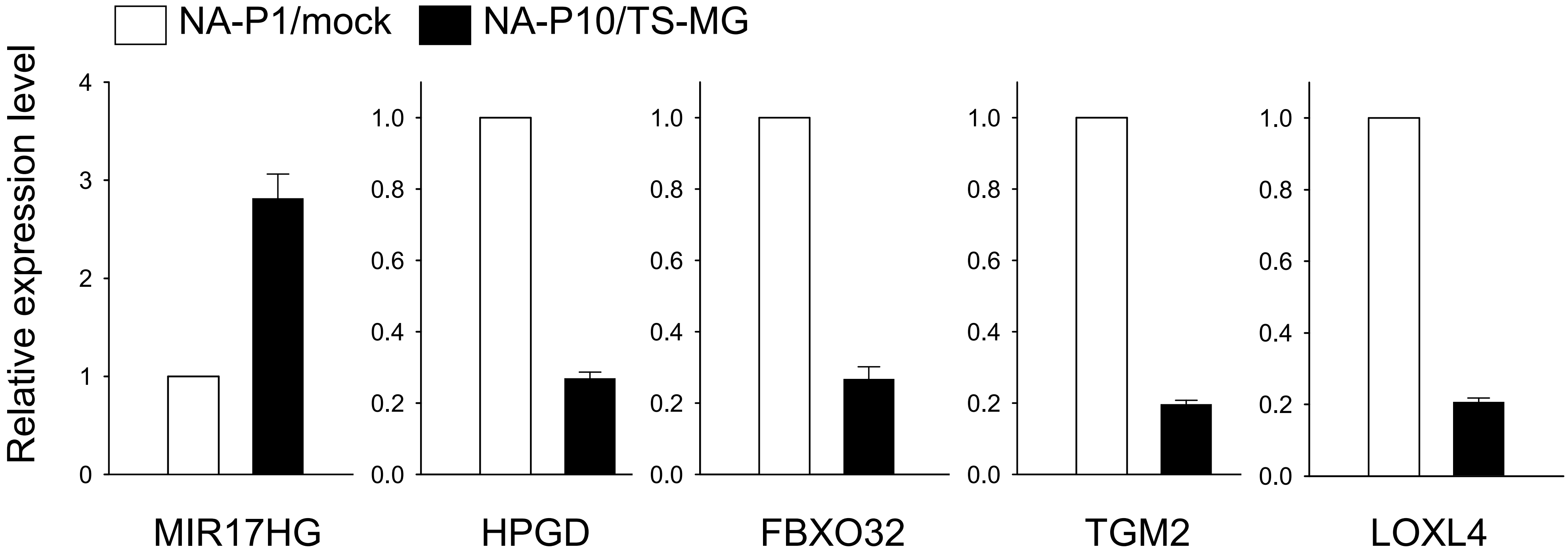

Figure S1. Quantitative RT-PCR validation of genes that were differentially displayed in NA-P1/mock and NA-P10/TS-MG cells. The expression level of gene in NA-P1/mock cells was adjusted as the base line (1-fold) and the relative expression level of gene in NA-P10/TS-MG cells was determined accordingly. Data indicate the mean expression level ± SD. MIR17HG: miR-17-92 cluster host gene; HPGD: hydroxyprostaglandin dehydrogenase 15-(NAD); FBXO32: f-box only protein 32; TGM2: transglutaminases 2; LOXL4: lysyl oxidase homolog 4.
