# Supplementary material for: The Synergistic Effect of Chemical Carcinogens Enhances Epstein-Barr Virus Reactivation and Tumor Progression of Nasopharyngeal Carcinoma Cells
Source: PLoS One. 2012 Sep 14;7(9):e44810. doi: 10.1371/journal.pone.0044810 (PMC3443098; doi:10.1371/journal.pone.0044810)
Supplement: Table S4 — Putative oncogenes and tumor suppressor genes that were altered after recurrent EBV reactivation. Several genes corresponding to the 170 probes in NA-P10/TS-MG cells are categorized as carcinogenesis-related oncogenes and tumor suppressor genes. Literatures from previous studies of other malignancies, indicating those genes as putative oncogenes/tumor suppressor genes, are indexed in this list. The alterations corresponded to the 26 genes of the 31 NPC biopsies are also noted. (PDF) [file pone.0044810.s005.pdf]

Table S4. Putative oncogenes/tumor suppressor genes derived from 170 probe sets.

A- Putative oncogenes upregulated in NA-P10/TS-MG when compared to NA-P1/mock cells

Gene symbol	Gene title	Location	Fold change	Reported upregulation in tumors[a.b]	Case of upregulation in 31 NPC biopsies[c]
POLQ	polymerase (DNA directed), theta	3q13.33	2.63	BC[1]	
SERPINB2	serpin peptidase inhibitor, clade B (ovalbumin), member 2	18q21.3	2.23	CC[2], EA[3]	
ODC1	ornithine decarboxylase 1	2p25	2.15	NB[4], PcC[5]	
KRT6A	keratin 6A	12q12-q13	2.10	BC[6]	
CCND2	cyclin D2	12p13	2.03	BL[7], BT[8], GC[9], L[10], TGCT[11], **NPC**[12]	24
ZNRF3	zinc and ring finger 3	22q12.1	2.02		25

B - Putative tumor suppressor genes down-regulated in NA-P10/TS-MG when compared to NA-P1/mock cells

Gene symbol	Gene title	Location	Fold change	Reported downregulation in tumors[a,b]	Case of downregulation in 31 NPC biopsies[c]
KYNU	kynureninase	2q22.2	-2.01	AML[13]	
TIMP2	TIMP metallopeptidase inhibitor 2	17q25	-2.02	CvC[14], NHL[15], PC[16]	
HISTH2BE	histone cluster 2, H2be	1q21-q23	-2.06	BC[17]	27
ASS1	argininosuccinate synthetase 1	9q34.1	-2.08	HCC[18], M[18], OC[19], OS[20], PC[21]	17
PSMB9	Proteasome (prosome, macropain) subunit, beta type, 9	6p21.3	-2.08	AML[22]	
LOXL2	lysyl oxidase-like 2	8p21.3-p21.2	-2.09	LC[23]	
GSTA4	glutathione S-transferase alpha 4	6p12.1	-2.11		
KLF9	Kruppel-like factor 9	9q13	-2.13	CC[24]	
PI3	peptidase inhibitor 3, skin-derived	20q12-q13	-2.14	EC[25], SCC[26], HNC[27]	28
CCNG2	cyclin G2	4q21.1	-2.18	GC[28], OC[29], TPC[30]	
ATP2B4	ATPase, Ca++ transporting, plasma membrane 4	1q32.1	-2.19	CC[31]	
HRK	harakiri, BCL2 interacting protein	12q24.22	-2.19	CC and GC[32], GB[33], PC[34], PCNSL[35]	
ZBTB10	zinc finger and BTB domain containing 10	8q13-q21.1	-2.22		
CXXC5	CXXC finger 5	5q31.2	-2.25	AML[36],	23
PRSS8	protease, serine, 8	16p11.2	-2.28	BC[37], BdC[38], PC[39]	
TSC22D3	TSC22 domain family, member 3	Xq22.3	-2.28	MM[40]	
PDZD2	PDZ domain containing 2	5p13.3	-2.32		23
IFITM1	interferon induced transmembrane protein 1	11p15.5	-2.36	CvC[41]	
SEPP1	selenoprotein P, plasma 1	5q31	-2.38	GC[42], PC[43]	
HPGD	hydroxyprostaglandin dehydrogenase 15-(NAD)	4q34-q35	-2.38	BC[44], CC[45], GC[46], LC[47], **NPC**[12]	
GBP1	guanylate binding protein 1	1p22.2	-2.40	BC[48], CC[49]	
TJP3	tight junction protein 3	19p13.3	-2.43		29
SCNN1A	sodium channel, nonvoltage-gated 1 alpha	12p13	-2.44	NB[50]	28
FBXO32	F-box protein 32	8q24.13	-2,46	OvC[51]	12
CDKN2B	cyclin-dependent kinase inhibitor 2B (p15, inhibits CDK4)	9p21	-2.46	ALL[52], NHL[53], **NPC**[12], TL[54]	16
SDPR	serum deprivation response	2q32-q33	-2.47	BC, PC, and RC[55]	
CYP1B1	cytochrome P450, family 1, subfamily B, polypeptide 1	2p21	-2.53	GC[56], OC[57]	
PDE5A	phosphodiesterase 5A, cGMP-specific	4q25-q27	-2.54	M[58]	
TGM2	transglutaminase 2	20q12	-2.65	BC[59], G[60]	
TXNIP	thioredoxin interacting protein	1q21.1	-2.67	BC[61], BdC[62], SCC[63]	12
LOXL4	lysyl oxidase-like 4	10q24	-2.73	BdC[64]	26
IL7	interleukin 7	8q12-q13	-2.78	PC[65]	
KLK10	kallikrein-related peptidase 10	19q13.3-q13.4	-2.84	BC[66], HCC[67], HNC[68], LC[69]	
INSIG1	insulin induced gene 1	7q36	-3.30	GC[70]	
KLK5	kallikrein-related peptidase 5	19q13.3-q13.4	-3.03	BC[71], PC[72]	
IRF9	interferon regulatory factor 9	14q11.2	-3.03		
GDF15	growth differentiation factor 15	19p13.11	-3.06	G[73]	20
C2 /// CFB	complement component 2 /// complement factor B	6p21.3	-3.11	**NPC**[74]	13
IFI27	interferon, alpha-inducible protein 27	14q32	-3.49		
FRMD3	FERM domain containing 3	9q21.32	-3.61	LC[75]	
KRT13	keratin 13	17q12-q21.2	-3.71	OC[76]	22
C15orf48	chromosome 15 open reading frame 48	15q21.1	-3.76	CvC[77], EC[78]	20
CLDN4	claudin 4	7q11.23	-3.78	BC[79], EC[80], PcC[81]	
AKR1B10	aldo-keto reductase family 1, member B10	7q33	-4.00	CC[82], HCC[83], CgC[84]	15
AKR1C1	aldo-keto reductase family 1, member C1	10p15-p14	-4.28	BC[85,86]	
AKR1C2	aldo-keto reductase family 1, member C2	10p15-p14	-4.35	BC[85,86]	18
AGR2	anterior gradient homolog 2 (Xenopus laevis)	7p21.3	-4.41	HCC[87], PC[88]	21

AKR1C3	aldo-keto reductase family 1, member C3	10p15-p14	-6.58	BC[86], CNT[89], EA[90]	22

C – Putative oncogenes that was **down-regulated** in NA-P10/TS-MG when compared to NA-P1/mock cells

Gene symbol	Gene title	Location	Fold change	Reported alteration in tumors[a.b]	Case of downregulation in 31 NPC biopsies[c]
MGLL	monoglyceride lipase	3q21.3	-2.12	UP CC[91], BC, M and OvC[92]	31
NEDD9	neural precursor cell expressed, developmentally down-regulated 9	6p25-p24	-2.16	UP BC[93], CC[94], HNC[95]<br>Down **NPC[96]**	
OSMR	oncostatin M receptor	5p13.1	-2.29	UP CvC[97], EC[98],<br>Down CC[99,100], PCNSL[101]	
ALDH3A1	aldehyde dehydrogenase 3 family, member A1	17p11.2	-2.33	UP HCC[102], LC[103], OC[104]	29
KLK8	kallikrein-related peptidase 8	19q13.3-q13.4	-2.54	UP HNC[105], OC[106]<br>Down OvC[107]	
L1CAM	L1 cell adhesion molecule	Xq28	-2.64	UP CC[108], OvC[109], RC[110]<br>Down MPNST[111]	
SERPINB3	serpin peptidase inhibitor, clade B (ovalbumin), member 3	18q21.3	-2.93	UP BC[112], HCC[113]<br>Down OC[114]	24
PSCA	prostate stem cell antigen	8q24.2	-4.55	UP BdC[115], PC[116,117], PcC[118]<br>Down EC and GC[119,120]	30
H19	H19, imprinted maternally expressed transcript	11p15.5	-4.62	UP BdC[121], EC[122], HNC[123], LC[124], PC[125]<br>Down HNC[123], **NPC**[12]	17
CCL5	chemokine (C-C motif) ligand 5	17q11.2-q12	-9.14	UP GC[126], PC[127]	

[a]: ALL: acute lymphoblastic leukemia; AML: acute myeloid leukemia; BC: breast carcinoma; BdC: bladder cancer; BL: B-cell lymphoma; BT: brain tumor; CC: colorectal cancer; CgC: cholangiocarcinoma; CvC: cervical cancer; CNT: central nervous tumors; EA: endometrial adenocarcinoma; EC: esophageal cancer; HCC: hepatocellular carcinoma; G: glioma; GB: glioblastomas; GC: gastric cancer; HNC: head and neck carcinoma; L: lymphoma; LC: lung cancer; M: melanoma; MM: multiple myeloma; NB: neuroblastoma; MPNST: malignant peripheral nerve sheath tumor; NPC: nasopharyngeal carcinoma; NHL: non-Hodgkin's lymphoma; OC: oral cancer; OvC: ovarian cancer; OS: osteosarcoma; PC: prostate cancer; PcC: pancreatic cancer; PCNSL: primary central nervous system lymphoma; RC: renal carcinoma; SCC: squamous cell carcinomas; TL: T-cell lymphoma; TGCT: Testicular germ cell tumors; TPC: thyroid papillary carcinoma.

[b]: References listed here were mainly focused on recent reports of clinical biopsy samples. For references listed in these tables, please refer to Document S1.

[c]: Upregulation of genes was defined when the expression level of NPC samples are above the 90th percentile of normal tissues; downregulation of genes was defined when the expression level of NPC samples are below the 10th percentile of normal tissues.
