# Supplementary material for: The Synergistic Effect of Chemical Carcinogens Enhances Epstein-Barr Virus Reactivation and Tumor Progression of Nasopharyngeal Carcinoma Cells
Source: PLoS One. 2012 Sep 14;7(9):e44810. doi: 10.1371/journal.pone.0044810 (PMC3443098; doi:10.1371/journal.pone.0044810)
Supplement: Table S5 — Differentially displayed genes in NA-P10/TS-MG cells arranged by the criteria of ten hallmarks of cancers. The genes from the 170 probe sets were categorized according to the criteria of ten hallmarks of cancers proposed by Hanahan and Weinberg [31]. Genes were categorized by their function and involved process provide by gene ontologies. (PDF) [file pone.0044810.s006.pdf]

Table S5. Differentially displayed genes in NA-P10/TS-MG cells arranged by the criteria of ten hallmarks of cancers*

Sustaining Proliferative Signaling	Evading Growth Suppressors	Resisting Cell Death	Enabling Replicative Immortality	Inducing Angiogenesis	Activating Invasion and Metastasis	Genome Instability and Mutation	Tumor-Promoting Inflammation	Reprogramming Energy Metabolism	Evading Immune Destruction
ODC1	TIMP2	SERPINB2		FGFBP1	TIMP2	POLQ	SGPP2[143]	ASS1	C1RL
KRT6A	FBXO2[129]	ODC1[132]		CCL2	MMP28	GSTA4	TSC22D3	MGLL	KLRC1/2
CCND2	HPGD	MX1		OSMR[97]	LOXL2	CXXC5[142]	GBP1[144]	LEPR	PSBM9
PSMB9	KLF9[130]	PSMB9		CYP1B1	GBP1[137]	ALDH3A1		ALDOC	IFITM1
CCNG2	IFITM1	PI3[133,134]		C3	NEDD9	SEPP1		ACSS2	CCL2
NEDD9	ZBTB10[131]	CARD16			TJP3	CYP1B1			OAS2
FGFBP1	OSMR[100]	ORAI3[135]			PRSS8[38]	TXNIP			TSC22D3
TJP3[128]	CTH	HRK			OSMR[97]	AKR1B10			C1S
OSMR	CDKN2B	CCL2			PDZD2	AKR1C1			HLA-F
ALDH3A1	TXNIP	PCSK9			BCAN	AKR1C2			IRF7
TFEC	LOXL4	ALDOC			GSN	ACSS2			GBP1[144]
NEDD9	KLK10	GSN			KLK6	AKR1C3			TXNIP
INSIG1	H19	FBXO32[51]			PDE5A[58]				TGM2
		KLK8			L1CAM				IL7[65]
		PDE5A			TGM2				C3
		TXNIP			LOXL4[138]				C2/CFB
		TGM2			NEDD9				OAS1
		IL7			KLK5[139]				
		SERPINB3			GDF15[140]				
		IRF9[136]			CLDN4				
		IFI27			AGR2[141]				
					NPNT				

*Genes were categorized by their function and involved process provide by (1) the Gene Ontology Annotation (UniProt-GOA), and/or (2) the gene summary provided by the Reference Sequence (RefSeq), National Center for Biotechnology Information, and/or (3) literatures from previous studies indicating these genes are involved in a specific process. By this principle, a single gene may be arranged into more than one group when it is involved in certain pathways. Underlined genes indicate genes that are upregulated, while others indicate genes that are downregulated in NA-P10/TS-MG cells. For references listed in this table, please refer to Document S1.
