# Supplementary material for: The Synergistic Effect of Chemical Carcinogens Enhances Epstein-Barr Virus Reactivation and Tumor Progression of Nasopharyngeal Carcinoma Cells
Source: PLoS One. 2012 Sep 14;7(9):e44810. doi: 10.1371/journal.pone.0044810 (PMC3443098; doi:10.1371/journal.pone.0044810)
Supplement: Table S6 — Primers used in semi-quantitative real-time PCR for genes of interest. (PDF) [file pone.0044810.s007.pdf]

Table S6. Primers used in semi-quantitative real-time PCR for genes of interest.

Gene	transcript_identifier	Fw_primer_sequence	Rev_primer_sequence
MIR17HG	Hs.24115	GCTGCTTGCAAAGTGTTGGTG	GGCTTGTAGTTAGACAGGTCTGC
LOXL4	Hs.306814	TGGGCCAACACCAACAGTTACG	AGGCCCTATCTGGCCATTTCTG
FBXO32	Hs.403933	GCAGCAGCAGCTGAACAACATTC	TAGGCACAAAGGCAGGTCAGTG
TGM2	Hs.517033	CTGGGCCTTTGTTTCCTTGTTGG	ACATGGAGTGGAGAGGATCCTTGG
HPGD	Hs.596913	TGTAAAGCTGCCCTGGATGAGC	AGCCACATCGCACTGGATGAAC

*Primers designed by QuantPrime at http://www.quantprime.de/.
