# Supplementary material for: The Synergistic Effect of Chemical Carcinogens Enhances Epstein-Barr Virus Reactivation and Tumor Progression of Nasopharyngeal Carcinoma Cells
Source: PLoS One. 2012 Sep 14;7(9):e44810. doi: 10.1371/journal.pone.0044810 (PMC3443098; doi:10.1371/journal.pone.0044810)
Supplement: Document S1 — List of cited references in Table S4 and S5. (PDF) [file pone.0044810.s008.pdf]

1. Lemee F, Bergoglio V, Fernandez-Vidal A, Machado-Silva A, Pillaire MJ, et al. (2010) DNA polymerase theta up-regulation is associated with poor survival in breast cancer, perturbs DNA replication, and promotes genetic instability. Proc Natl Acad Sci U S A 107: 13390-13395.
2. Halamkova J, Kiss I, Pavlovsky Z, Tomasek J, Jarkovsky J, et al. (2011) Clinical significance of the plasminogen activator system in relation to grade of tumor and treatment response in colorectal carcinoma patients. Neoplasma 58: 377-385.
3. Osmak M, Babic D, Abramic M, Milicic D, Vrhovec I, et al. (2001) Plasminogen activator inhibitor type 2: potential prognostic factor for endometrial carcinomas. Neoplasma 48: 462-467.
4. Hogarty MD, Norris MD, Davis K, Liu X, Evageliou NF, et al. (2008) ODC1 is a critical determinant of MYCN oncogenesis and a therapeutic target in neuroblastoma. Cancer Res 68: 9735-9745.
5. Subhi AL, Tang B, Balsara BR, Altomare DA, Testa JR, et al. (2004) Loss of methylthioadenosine phosphorylase and elevated ornithine decarboxylase is common in pancreatic cancer. Clin Cancer Res 10: 7290-7296.
6. Tischkowitz M, Brunet JS, Begin LR, Huntsman DG, Cheang MC, et al. (2007) Use of immunohistochemical markers can refine prognosis in triple negative breast cancer. BMC Cancer 7: 134.
7. Delmer A, Ajchenbaum-Cymbalista F, Tang R, Ramond S, Faussat AM, et al. (1995) Overexpression of cyclin D2 in chronic B-cell malignancies. Blood 85: 2870-2876.
8. Kheirollahi M, Mehr-Azin M, Kamalian N, Mehdipour P (2011) Expression of cyclin D2, P53, Rb and ATM cell cycle genes in brain tumors. Med Oncol 28: 7-14.
9. Takano Y, Kato Y, Masuda M, Ohshima Y, Okayasu I (1999) Cyclin D2, but not cyclin D1, overexpression closely correlates with gastric cancer progression and prognosis. J Pathol 189: 194-200.
10. Igawa T, Sato Y, Takata K, Fushimi S, Tamura M, et al. (2011) Cyclin D2 is overexpressed in proliferation centers of chronic lymphocytic leukemia/small lymphocytic lymphoma. Cancer Sci 102: 2103-2107.
11. Schmidt BA, Rose A, Steinhoff C, Strohmeyer T, Hartmann M, et al. (2001) Up-regulation of cyclin-dependent kinase 4/cyclin D2 expression but down-regulation of cyclin-dependent kinase 2/cyclin E in testicular germ cell tumors. Cancer Res 61: 4214-4221.
12. Hu C (2010) Genetic and gene expression analysis of nasopharyngeal carcinoma (NPC). http://etheses.bham.ac.uk/1063/: Ph.D. thesis, University of

Birmingham.
13. Marcucci G, Maharry K, Wu YZ, Radmacher MD, Mrozek K, et al. (2010) IDH1 and IDH2 gene mutations identify novel molecular subsets within de novo cytogenetically normal acute myeloid leukemia: a Cancer and Leukemia Group B study. J Clin Oncol 28: 2348-2355.
14. Ivanova T, Vinokurova S, Petrenko A, Eshilev E, Solovyova N, et al. (2004) Frequent hypermethylation of 5' flanking region of TIMP-2 gene in cervical cancer. Int J Cancer 108: 882-886.
15. Galm O, Suzuki H, Akiyama Y, Esteller M, Brock MV, et al. (2005) Inactivation of the tissue inhibitor of metalloproteinases-2 gene by promoter hypermethylation in lymphoid malignancies. Oncogene 24: 4799-4805.
16. Pulukuri SM, Patibandla S, Patel J, Estes N, Rao JS (2007) Epigenetic inactivation of the tissue inhibitor of metalloproteinase-2 (TIMP-2) gene in human prostate tumors. Oncogene 26: 5229-5237.
17. Prenzel T, Begus-Nahrmann Y, Kramer F, Hennion M, Hsu C, et al. (2011) Estrogen-dependent gene transcription in human breast cancer cells relies upon proteasome-dependent monoubiquitination of histone H2B. Cancer Res 71: 5739-5753.
18. Ensor CM, Holtsberg FW, Bomalaski JS, Clark MA (2002) Pegylated arginine deiminase (ADI-SS PEG20,000 mw) inhibits human melanomas and hepatocellular carcinomas in vitro and in vivo. Cancer Res 62: 5443-5450.
19. Szlosarek PW, Grimshaw MJ, Wilbanks GD, Hagemann T, Wilson JL, et al. (2007) Aberrant regulation of argininosuccinate synthetase by TNF-alpha in human epithelial ovarian cancer. Int J Cancer 121: 6-11.
20. Kobayashi E, Masuda M, Nakayama R, Ichikawa H, Satow R, et al. (2010) Reduced argininosuccinate synthetase is a predictive biomarker for the development of pulmonary metastasis in patients with osteosarcoma. Mol Cancer Ther 9: 535-544.
21. Kim RH, Coates JM, Bowles TL, McNerney GP, Sutcliffe J, et al. (2009) Arginine deiminase as a novel therapy for prostate cancer induces autophagy and caspase-independent apoptosis. Cancer Res 69: 700-708.
22. Hoves S, Aigner M, Pfeiffer C, Laumer M, Obermann EC, et al. (2009) In situ analysis of the antigen-processing machinery in acute myeloid leukaemic blasts by tissue microarray. Leukemia 23: 877-885.
23. Zhan P, Shen XK, Qian Q, Zhu JP, Zhang Y, et al. (2011) Down-regulation of lysyl oxidase-like 2 (LOXL2) is associated with disease progression in lung adenocarcinomas. Med Oncol.
24. Kang L, Lu B, Xu J, Hu H, Lai M (2008) Downregulation of Kruppel-like factor 9

in human colorectal cancer. Pathol Int 58: 334-338.
25. Yamamoto S, Egami H, Kurizaki T, Ohmachi H, Hayashi N, et al. (1997) Immunohistochemical expression of SKALP/elafin in squamous cell carcinoma of the oesophagus. Br J Cancer 76: 1081-1086.
26. Alkemade HA, van Vlijmen-Willems IM, van Haelst UJ, van de Kerkhof PC, Schalkwijk J (1994) Demonstration of skin-derived antileukoproteinase (SKALP) and its target enzyme human leukocyte elastase in squamous cell carcinoma. J Pathol 174: 121-129.
27. Westin U, Nystrom M, Ljungcrantz I, Eriksson B, Ohlsson K (2002) The presence of elafin, SLPI, IL1-RA and STNFalpha RI in head and neck squamous cell carcinomas and their relation to the degree of tumour differentiation. Mediators Inflamm 11: 7-12.
28. Choi MG, Noh JH, An JY, Hong SK, Park SB, et al. (2009) Expression levels of cyclin G2, but not cyclin E, correlate with gastric cancer progression. J Surg Res 157: 168-174.
29. Kim Y, Shintani S, Kohno Y, Zhang R, Wong DT (2004) Cyclin G2 dysregulation in human oral cancer. Cancer Res 64: 8980-8986.
30. Ito Y, Yoshida H, Uruno T, Nakano K, Takamura Y, et al. (2003) Decreased expression of cyclin G2 is significantly linked to the malignant transformation of papillary carcinoma of the thyroid. Anticancer Res 23: 2335-2338.
31. Aung CS, Ye W, Plowman G, Peters AA, Monteith GR, et al. (2009) Plasma membrane calcium ATPase 4 and the remodeling of calcium homeostasis in human colon cancer cells. Carcinogenesis 30: 1962-1969.
32. Obata T, Toyota M, Satoh A, Sasaki Y, Ogi K, et al. (2003) Identification of HRK as a target of epigenetic inactivation in colorectal and gastric cancer. Clin Cancer Res 9: 6410-6418.
33. Nakamura M, Ishida E, Shimada K, Nakase H, Sakaki T, et al. (2005) Frequent HRK inactivation associated with low apoptotic index in secondary glioblastomas. Acta Neuropathol 110: 402-410.
34. Higuchi T, Nakamura M, Shimada K, Ishida E, Hirao K, et al. (2008) HRK inactivation associated with promoter methylation and LOH in prostate cancer. Prostate 68: 105-113.
35. Nakamura M, Shimada K, Konishi N (2008) The role of HRK gene in human cancer. Oncogene 27 Suppl 1: S105-113.
36. Pendino F, Nguyen E, Jonassen I, Dysvik B, Azouz A, et al. (2009) Functional involvement of RINF, retinoid-inducible nuclear factor (CXXC5), in normal and tumoral human myelopoiesis. Blood 113: 3172-3181.
37. Chen LM, Chai KX (2002) Prostasin serine protease inhibits breast cancer

invasiveness and is transcriptionally regulated by promoter DNA methylation. Int J Cancer 97: 323-329.
38. Chen LM, Verity NJ, Chai KX (2009) Loss of prostasin (PRSS8) in human bladder transitional cell carcinoma cell lines is associated with epithelial-mesenchymal transition (EMT). BMC Cancer 9: 377.
39. Takahashi S, Suzuki S, Inaguma S, Ikeda Y, Cho YM, et al. (2003) Down-regulated expression of prostasin in high-grade or hormone-refractory human prostate cancers. Prostate 54: 187-193.
40. Grugan KD, Ma C, Singhal S, Krett NL, Rosen ST (2008) Dual regulation of glucocorticoid-induced leucine zipper (GILZ) by the glucocorticoid receptor and the PI3-kinase/AKT pathways in multiple myeloma. J Steroid Biochem Mol Biol 110: 244-254.
41. Pan Z, Chen S, Pan X, Wang Z, Han H, et al. (2010) Differential gene expression identified in Uigur women cervical squamous cell carcinoma by suppression subtractive hybridization. Neoplasma 57: 123-128.
42. Wang Q, Gong L, Dong R, Qiao Q, He XL, et al. (2009) Tissue microarray assessment of selenoprotein P expression in gastric adenocarcinoma. J Int Med Res 37: 169-174.
43. Gonzalez-Moreno O, Boque N, Redrado M, Milagro F, Campion J, et al. Selenoprotein-P is down-regulated in prostate cancer, which results in lack of protection against oxidative damage. Prostate.
44. Wolf I, O'Kelly J, Rubinek T, Tong M, Nguyen A, et al. (2006) 15-hydroxyprostaglandin dehydrogenase is a tumor suppressor of human breast cancer. Cancer Res 66: 7818-7823.
45. Backlund MG, Mann JR, Holla VR, Buchanan FG, Tai HH, et al. (2005) 15-Hydroxyprostaglandin dehydrogenase is down-regulated in colorectal cancer. J Biol Chem 280: 3217-3223.
46. Liu Z, Wang X, Lu Y, Han S, Zhang F, et al. (2008) Expression of 15-PGDH is downregulated by COX-2 in gastric cancer. Carcinogenesis 29: 1219-1227.
47. Ding Y, Tong M, Liu S, Moscow JA, Tai HH (2005) NAD+-linked 15-hydroxyprostaglandin dehydrogenase (15-PGDH) behaves as a tumor suppressor in lung cancer. Carcinogenesis 26: 65-72.
48. Ascierto ML, Kmieciak M, Idowu MO, Manjili R, Zhao Y, et al. A signature of immune function genes associated with recurrence-free survival in breast cancer patients. Breast Cancer Res Treat.
49. Naschberger E, Croner RS, Merkel S, Dimmler A, Tripal P, et al. (2008) Angiostatic immune reaction in colorectal carcinoma: Impact on survival and perspectives for antiangiogenic therapy. Int J Cancer 123: 2120-2129.

50. Caren H, Djos A, Nethander M, Sjoberg RM, Kogner P, et al. (2011) Identification of epigenetically regulated genes that predict patient outcome in neuroblastoma. BMC Cancer 11: 66.
51. Chou JL, Su HY, Chen LY, Liao YP, Hartman-Frey C, et al. (2010) Promoter hypermethylation of FBXO32, a novel TGF-beta/SMAD4 target gene and tumor suppressor, is associated with poor prognosis in human ovarian cancer. Lab Invest 90: 414-425.
52. Gardiner RB, Morash BA, Riddell C, Wang H, Fernandez CV, et al. (2011) Using MS-MLPA as an efficient screening tool for detecting 9p21 abnormalities in pediatric acute lymphoblastic leukemia. Pediatr Blood Cancer.
53. Guney S, Bertrand P, Jardin F, Ruminy P, Kerckaert JP, et al. (2011) Molecular characterization of 9p21 deletions shows a minimal common deleted region removing CDKN2A exon 1 and CDKN2B exon 2 in diffuse large B-cell lymphomas. Genes Chromosomes Cancer 50: 715-725.
54. Krieger D, Moericke A, Oschlies I, Zimmermann M, Schrappe M, et al. (2010) Frequency and clinical relevance of DNA microsatellite alterations of the CDKN2A/B, ATM and p53 gene loci: a comparison between pediatric precursor T-cell lymphoblastic lymphoma and T-cell lymphoblastic leukemia. Haematologica 95: 158-162.
55. Li X, Jia Z, Shen Y, Ichikawa H, Jarvik J, et al. (2008) Coordinate suppression of Sdpr and Fhl1 expression in tumors of the breast, kidney, and prostate. Cancer Sci 99: 1326-1333.
56. Hiraki M, Kitajima Y, Koga Y, Tanaka T, Nakamura J, et al. (2011) Aberrant gene methylation is a biomarker for the detection of cancer cells in peritoneal wash samples from advanced gastric cancer patients. Ann Surg Oncol 18: 3013-3019.
57. Pradhan S, Nagashri MN, Gopinath KS, Kumar A (2011) Expression profiling of CYP1B1 in oral squamous cell carcinoma: counterintuitive downregulation in tumors. PLoS One 6: e27914.
58. Arozarena I, Sanchez-Laorden B, Packer L, Hidalgo-Carcedo C, Hayward R, et al. (2011) Oncogenic BRAF induces melanoma cell invasion by downregulating the cGMP-specific phosphodiesterase PDE5A. Cancer Cell 19: 45-57.
59. Ai L, Kim WJ, Demircan B, Dyer LM, Bray KJ, et al. (2008) The transglutaminase 2 gene (TGM2), a potential molecular marker for chemotherapeutic drug sensitivity, is epigenetically silenced in breast cancer. Carcinogenesis 29: 510-518.
60. Dyer LM, Schooler KP, Ai L, Klop C, Qiu J, et al. (2011) The transglutaminase 2 gene is aberrantly hypermethylated in glioma. J Neurooncol 101: 429-440.

61. Yan GR, Xu SH, Tan ZL, Liu L, He QY (2011) Global identification of miR-373-regulated genes in breast cancer by quantitative proteomics. Proteomics 11: 912-920.
62. Nishizawa K, Nishiyama H, Matsui Y, Kobayashi T, Saito R, et al. (2011) Thioredoxin-interacting protein suppresses bladder carcinogenesis. Carcinogenesis 32: 1459-1466.
63. Shin KH, Kim RH, Kang MK, Park NH (2008) hnRNP G elicits tumor-suppressive activity in part by upregulating the expression of Txnip. Biochem Biophys Res Commun 372: 880-885.
64. Wu G, Guo Z, Chang X, Kim MS, Nagpal JK, et al. (2007) LOXL1 and LOXL4 are epigenetically silenced and can inhibit ras/extracellular signal-regulated kinase signaling pathway in human bladder cancer. Cancer Res 67: 4123-4129.
65. Di Carlo E, D'Antuono T, Pompa P, Giuliani R, Rosini S, et al. (2009) The lack of epithelial interleukin-7 and BAFF/BLyS gene expression in prostate cancer as a possible mechanism of tumor escape from immunosurveillance. Clin Cancer Res 15: 2979-2987.
66. Kioulafa M, Kaklamanis L, Stathopoulos E, Mavroudis D, Georgoulias V, et al. (2009) Kallikrein 10 (KLK10) methylation as a novel prognostic biomarker in early breast cancer. Ann Oncol 20: 1020-1025.
67. Lu CY, Hsieh SY, Lu YJ, Wu CS, Chen LC, et al. (2009) Aberrant DNA methylation profile and frequent methylation of KLK10 and OXGR1 genes in hepatocellular carcinoma. Genes Chromosomes Cancer 48: 1057-1068.
68. Worsham MJ, Chen KM, Meduri V, Nygren AO, Errami A, et al. (2006) Epigenetic events of disease progression in head and neck squamous cell carcinoma. Arch Otolaryngol Head Neck Surg 132: 668-677.
69. Zhang Y, Song H, Miao Y, Wang R, Chen L (2010) Frequent transcriptional inactivation of Kallikrein 10 gene by CpG island hypermethylation in non-small cell lung cancer. Cancer Sci 101: 934-940.
70. Kaneda A, Kaminishi M, Nakanishi Y, Sugimura T, Ushijima T (2002) Reduced expression of the insulin-induced protein 1 and p41 Arp2/3 complex genes in human gastric cancers. Int J Cancer 100: 57-62.
71. Li X, Liu J, Wang Y, Zhang L, Ning L, et al. (2009) Parallel underexpression of kallikrein 5 and kallikrein 7 mRNA in breast malignancies. Cancer Sci 100: 601-607.
72. Korbakis D, Gregorakis AK, Scorilas A (2009) Quantitative analysis of human kallikrein 5 (KLK5) expression in prostate needle biopsies: an independent cancer biomarker. Clin Chem 55: 904-913.
73. Kadowaki M, Yoshioka H, Kamitani H, Watanabe T, Wade PA, et al. (2012) DNA

methylation-mediated silencing of nonsteroidal anti-inflammatory drug-activated gene (NAG-1/GDF15) in glioma cell lines. Int J Cancer 130: 267-277.
74. Seriramalu R, Pang WW, Jayapalan JJ, Mohamed E, Abdul-Rahman PS, et al. (2010) Application of champedak mannose-binding lectin in the glycoproteomic profiling of serum samples unmasks reduced expression of alpha-2 macroglobulin and complement factor B in patients with nasopharyngeal carcinoma. Electrophoresis 31: 2388-2395.
75. Haase D, Meister M, Muley T, Hess J, Teurich S, et al. (2007) FRMD3, a novel putative tumour suppressor in NSCLC. Oncogene 26: 4464-4468.
76. Mikami T, Cheng J, Maruyama S, Kobayashi T, Funayama A, et al. (2011) Emergence of keratin 17 vs. loss of keratin 13: their reciprocal immunohistochemical profiles in oral carcinoma in situ. Oral Oncol 47: 497-503.
77. Sova P, Feng Q, Geiss G, Wood T, Strauss R, et al. (2006) Discovery of novel methylation biomarkers in cervical carcinoma by global demethylation and microarray analysis. Cancer Epidemiol Biomarkers Prev 15: 114-123.
78. Zhou J, Wang H, Lu A, Hu G, Luo A, et al. (2002) A novel gene, NMES1, downregulated in human esophageal squamous cell carcinoma. Int J Cancer 101: 311-316.
79. Tokes AM, Kulka J, Paku S, Szik A, Paska C, et al. (2005) Claudin-1, -3 and -4 proteins and mRNA expression in benign and malignant breast lesions: a research study. Breast Cancer Res 7: R296-305.
80. Sung CO, Han SY, Kim SH (2011) Low expression of claudin-4 is associated with poor prognosis in esophageal squamous cell carcinoma. Ann Surg Oncol 18: 273-281.
81. Tsutsumi K, Sato N, Tanabe R, Mizumoto K, Morimatsu K, et al. (2011) Claudin-4 Expression Predicts Survival in Pancreatic Ductal Adenocarcinoma. Ann Surg Oncol.
82. Kropotova ES, Tychko RA, Zinov'eva OL, Zyrianova AF, Khankin SL, et al. (2010) [Downregulation of AKR1B10 gene expression in colorectal cancer]. Mol Biol (Mosk) 44: 243-250.
83. Schmitz KJ, Sotiropoulos GC, Baba HA, Schmid KW, Muller D, et al. (2011) AKR1B10 expression is associated with less aggressive hepatocellular carcinoma: a clinicopathological study of 168 cases. Liver Int 31: 810-816.
84. Heringlake S, Hofdmann M, Fiebeler A, Manns MP, Schmiegel W, et al. (2010) Identification and expression analysis of the aldo-ketoreductase1-B10 gene in primary malignant liver tumours. J Hepatol 52: 220-227.

85. Ji Q, Aoyama C, Nien YD, Liu PI, Chen PK, et al. (2004) Selective loss of AKR1C1 and AKR1C2 in breast cancer and their potential effect on progesterone signaling. Cancer Res 64: 7610-7617.
86. Lewis MJ, Wiebe JP, Heathcote JG (2004) Expression of progesterone metabolizing enzyme genes (AKR1C1, AKR1C2, AKR1C3, SRD5A1, SRD5A2) is altered in human breast carcinoma. BMC Cancer 4: 27.
87. Lepreux S, Bioulac-Sage P, Chevet E (2011) Differential expression of the anterior gradient protein-2 is a conserved feature during morphogenesis and carcinogenesis of the biliary tree. Liver Int 31: 322-328.
88. Maresh EL, Mah V, Alavi M, Horvath S, Bagryanova L, et al. (2010) Differential expression of anterior gradient gene AGR2 in prostate cancer. BMC Cancer 10: 680.
89. Park AL, Lin HK, Yang Q, Sing CW, Fan M, et al. (2010) Differential expression of type 2 3alpha/type 5 17beta-hydroxysteroid dehydrogenase (AKR1C3) in tumors of the central nervous system. Int J Clin Exp Pathol 3: 743-754.
90. Zakharov V, Lin HK, Azzarello J, McMeekin S, Moore KN, et al. (2010) Suppressed expression of type 2 3alpha/type 5 17beta-hydroxysteroid dehydrogenase (AKR1C3) in endometrial hyperplasia and carcinoma. Int J Clin Exp Pathol 3: 608-617.
91. Ye L, Zhang B, Seviour EG, Tao KX, Liu XH, et al. (2011) Monoacylglycerol lipase (MAGL) knockdown inhibits tumor cells growth in colorectal cancer. Cancer Lett 307: 6-17.
92. Nomura DK, Long JZ, Niessen S, Hoover HS, Ng SW, et al. (2010) Monoacylglycerol lipase regulates a fatty acid network that promotes cancer pathogenesis. Cell 140: 49-61.
93. Kong C, Wang C, Wang L, Ma M, Niu C, et al. (2011) NEDD9 is a positive regulator of epithelial-mesenchymal transition and promotes invasion in aggressive breast cancer. PLoS One 6: e22666.
94. Li Y, Bavarva JH, Wang Z, Guo J, Qian C, et al. (2011) HEF1, a novel target of Wnt signaling, promotes colonic cell migration and cancer progression. Oncogene 30: 2633-2643.
95. Lucas JT, Jr., Salimath BP, Slomiany MG, Rosenzweig SA (2010) Regulation of invasive behavior by vascular endothelial growth factor is HEF1-dependent. Oncogene 29: 4449-4459.
96. Li Y, Fu L, Wong AM, Fan YH, Li MX, et al. (2011) Identification of genes with allelic imbalance on 6p associated with nasopharyngeal carcinoma in southern Chinese. PLoS One 6: e14562.
97. Winder DM, Chattopadhyay A, Muralidhar B, Bauer J, English WR, et al. (2011)

Overexpression of the oncostatin M receptor in cervical squamous cell carcinoma cells is associated with a pro-angiogenic phenotype and increased cell motility and invasiveness. J Pathol 225: 448-462.
98. Kausar T, Sharma R, Hasan MR, Saraya A, Chattopadhyay TK, et al. (2011) Overexpression of a splice variant of oncostatin M receptor beta in human esophageal squamous carcinoma. Cell Oncol (Dordr) 34: 177-187.
99. Hibi K, Goto T, Sakuraba K, Shirahata A, Saito M, et al. (2011) Methylation of OSMR gene is frequently observed in non-invasive colorectal cancer. Anticancer Res 31: 1293-1295.
100. Kim MS, Louwagie J, Carvalho B, Terhaar Sive Droste JS, Park HL, et al. (2009) Promoter DNA methylation of oncostatin m receptor-beta as a novel diagnostic and therapeutic marker in colon cancer. PLoS One 4: e6555.
101. Sung CO, Kim SC, Karnan S, Karube K, Shin HJ, et al. (2011) Genomic profiling combined with gene expression profiling in primary central nervous system lymphoma. Blood 117: 1291-1300.
102. Chang C, Hsu LC, Dave V, Yoshida A (1998) Expression of human aldehyde dehydrogenase-3 associated with hepatocellular carcinoma: promoter regions and nuclear protein factors related to the expression. Int J Mol Med 2: 333-338.
103. Patel M, Lu L, Zander DS, Sreerama L, Coco D, et al. (2008) ALDH1A1 and ALDH3A1 expression in lung cancers: correlation with histologic type and potential precursors. Lung Cancer 59: 340-349.
104. Giebultowicz J, Wroczynski P, Piekarczyk J, Wierzchowski J (2008) Fluorimetric detection of aldehyde dehydrogenase activity in human tissues in diagnostic of cancers of oral cavity. Acta Pol Pharm 65: 81-84.
105. Darling MR, Tsai S, Jackson-Boeters L, Daley TD, Diamandis EP (2008) Human kallikrein 8 expression in salivary gland tumors. Head Neck Pathol 2: 169-174.
106. Pettus JR, Johnson JJ, Shi Z, Davis JW, Koblinski J, et al. (2009) Multiple kallikrein (KLK 5, 7, 8, and 10) expression in squamous cell carcinoma of the oral cavity. Histol Histopathol 24: 197-207.
107. Shigemasa K, Tian X, Gu L, Tanimoto H, Underwood LJ, et al. (2004) Human kallikrein 8 (hK8/TADG-14) expression is associated with an early clinical stage and favorable prognosis in ovarian cancer. Oncol Rep 11: 1153-1159.
108. Kajiwara Y, Ueno H, Hashiguchi Y, Shinto E, Shimazaki H, et al. (2011) Expression of l1 cell adhesion molecule and morphologic features at the invasive front of colorectal cancer. Am J Clin Pathol 136: 138-144.
109. Hauser S, Bickel L, Weinspach D, Gerg M, Schafer MK, et al. (2011) Full-length

L1CAM and not its Delta2Delta27 splice variant promotes metastasis through induction of gelatinase expression. PLoS One 6: e18989.
110. Doberstein K, Wieland A, Lee SB, Blaheta RA, Wedel S, et al. (2011) L1-CAM expression in ccRCC correlates with shorter patients survival times and confers chemoresistance in renal cell carcinoma cells. Carcinogenesis 32: 262-270.
111. Blessmann M, Grobe A, Quaas A, Kaifi JT, Mistakidis G, et al. (2011) Adhesion molecule L1 is down-regulated in malignant peripheral nerve sheath tumors versus benign neurofibromatosis type 1-associated tumors. Oral Surg Oral Med Oral Pathol Oral Radiol Endod.
112. Catanzaro JM, Guerriero JL, Liu J, Ullman E, Sheshadri N, et al. (2011) Elevated expression of squamous cell carcinoma antigen (SCCA) is associated with human breast carcinoma. PLoS One 6: e19096.
113. Pontisso P, Calabrese F, Benvegnu L, Lise M, Belluco C, et al. (2004) Overexpression of squamous cell carcinoma antigen variants in hepatocellular carcinoma. Br J Cancer 90: 833-837.
114. Shiiba M, Nomura H, Shinozuka K, Saito K, Kouzu Y, et al. (2010) Down-regulated expression of SERPIN genes located on chromosome 18q21 in oral squamous cell carcinomas. Oncol Rep 24: 241-249.
115. Amara N, Palapattu GS, Schrage M, Gu Z, Thomas GV, et al. (2001) Prostate stem cell antigen is overexpressed in human transitional cell carcinoma. Cancer Res 61: 4660-4665.
116. Barbisan F, Mazzucchelli R, Santinelli A, Scarpelli M, Lopez-Beltran A, et al. (2010) Expression of prostate stem cell antigen in high-grade prostatic intraepithelial neoplasia and prostate cancer. Histopathology 57: 572-579.
117. Zhigang Z, Wenlv S (2004) Prostate stem cell antigen (PSCA) expression in human prostate cancer tissues: implications for prostate carcinogenesis and progression of prostate cancer. Jpn J Clin Oncol 34: 414-419.
118. Wente MN, Jain A, Kono E, Berberat PO, Giese T, et al. (2005) Prostate stem cell antigen is a putative target for immunotherapy in pancreatic cancer. Pancreas 31: 119-125.
119. Bahrenberg G, Brauers A, Joost HG, Jakse G (2000) Reduced expression of PSCA, a member of the LY-6 family of cell surface antigens, in bladder, esophagus, and stomach tumors. Biochem Biophys Res Commun 275: 783-788.
120. Sakamoto H, Yoshimura K, Saeki N, Katai H, Shimoda T, et al. (2008) Genetic variation in PSCA is associated with susceptibility to diffuse-type gastric cancer. Nat Genet 40: 730-740.

121. Amit D, Hochberg A (2010) Development of targeted therapy for bladder cancer mediated by a double promoter plasmid expressing diphtheria toxin under the control of H19 and IGF2-P4 regulatory sequences. J Transl Med 8: 134.
122. Hibi K, Nakamura H, Hirai A, Fujikake Y, Kasai Y, et al. (1996) Loss of H19 imprinting in esophageal cancer. Cancer Res 56: 480-482.
123. Mirisola V, Mora R, Esposito AI, Guastini L, Tabacchiera F, et al. (2011) A prognostic multigene classifier for squamous cell carcinomas of the larynx. Cancer Lett 307: 37-46.
124. Kondo M, Suzuki H, Ueda R, Osada H, Takagi K, et al. (1995) Frequent loss of imprinting of the H19 gene is often associated with its overexpression in human lung cancers. Oncogene 10: 1193-1198.
125. Scaiewicz V, Sorin V, Fellig Y, Birman T, Mizrahi A, et al. (2010) Use of H19 Gene Regulatory Sequences in DNA-Based Therapy for Pancreatic Cancer. J Oncol 2010: 178174.
126. Park JY, Park KH, Bang S, Kim MH, Lee JE, et al. (2007) CXCL5 overexpression is associated with late stage gastric cancer. J Cancer Res Clin Oncol 133: 835-840.
127. Li A, King J, Moro A, Sugi MD, Dawson DW, et al. (2011) Overexpression of CXCL5 is associated with poor survival in patients with pancreatic cancer. Am J Pathol 178: 1340-1349.
128. Capaldo CT, Koch S, Kwon M, Laur O, Parkos CA, et al. (2011) Tight function zonula occludens-3 regulates cyclin D1-dependent cell proliferation. Mol Biol Cell 22: 1677-1685.
129. Wen H, Kim N, Fuentes EJ, Mallinger A, Gonzalez-Alegre P, et al. (2010) FBG1 is a promiscuous ubiquitin ligase that sequesters APC2 and causes S-phase arrest. Cell Cycle 9: 4506-4517.
130. Ying M, Sang Y, Li Y, Guerrero-Cazares H, Quinones-Hinojosa A, et al. (2011) Kruppel-like family of transcription factor 9, a differentiation-associated transcription factor, suppresses Notch1 signaling and inhibits glioblastoma-initiating stem cells. Stem Cells 29: 20-31.
131. Li X, Mertens-Talcott SU, Zhang S, Kim K, Ball J, et al. (2010) MicroRNA-27a Indirectly Regulates Estrogen Receptor {alpha} Expression and Hormone Responsiveness in MCF-7 Breast Cancer Cells. Endocrinology 151: 2462-2473.
132. Hsieh WC, Hsu PC, Liao YF, Young ST, Wang ZW, et al. (2010) Overexpression of ornithine decarboxylase suppresses thapsigargin-induced apoptosis. Mol Cells 30: 311-318.
133. Caruso JA, Hunt KK, Keyomarsi K (2010) The neutrophil elastase inhibitor

elafin triggers rb-mediated growth arrest and caspase-dependent apoptosis in breast cancer. Cancer Res 70: 7125-7136.
134. Yu KS, Lee Y, Kim CM, Park EC, Choi J, et al. (2010) The protease inhibitor, elafin, induces p53-dependent apoptosis in human melanoma cells. Int J Cancer 127: 1308-1320.
135. Faouzi M, Hague F, Potier M, Ahidouch A, Sevestre H, et al. (2011) Down-regulation of Orai3 arrests cell-cycle progression and induces apoptosis in breast cancer cells but not in normal breast epithelial cells. J Cell Physiol 226: 542-551.
136. Tsuno T, Mejido J, Zhao T, Schmeisser H, Morrow A, et al. (2009) IRF9 is a key factor for eliciting the antiproliferative activity of IFN-alpha. J Immunother 32: 803-816.
137. Hammon M, Herrmann M, Bleiziffer O, Pryymachuk G, Andreoli L, et al. (2011) Role of guanylate binding protein-1 in vascular defects associated with chronic inflammatory diseases. J Cell Mol Med 15: 1582-1592.
138. Kim DJ, Lee DC, Yang SJ, Lee JJ, Bae EM, et al. (2008) Lysyl oxidase like 4, a novel target gene of TGF-beta1 signaling, can negatively regulate TGF-beta1-induced cell motility in PLC/PRF/5 hepatoma cells. Biochem Biophys Res Commun 373: 521-527.
139. Jiang R, Shi Z, Johnson JJ, Liu Y, Stack MS (2011) Kallikrein-5 promotes cleavage of desmoglein-1 and loss of cell-cell cohesion in oral squamous cell carcinoma. J Biol Chem 286: 9127-9135.
140. Cheng JC, Chang HM, Leung PC (2011) Wild-type p53 attenuates cancer cell motility by inducing growth differentiation factor-15 expression. Endocrinology 152: 2987-2995.
141. Park K, Chung YJ, So H, Kim K, Park J, et al. (2011) AGR2, a mucinous ovarian cancer marker, promotes cell proliferation and migration. Exp Mol Med 43: 91-100.
142. Zhang M, Wang R, Wang Y, Diao F, Lu F, et al. (2009) The CXXC finger 5 protein is required for DNA damage-induced p53 activation. Sci China C Life Sci 52: 528-538.
143. Mechtcheriakova D, Wlachos A, Sobanov J, Kopp T, Reuschel R, et al. (2007) Sphingosine 1-phosphate phosphatase 2 is induced during inflammatory responses. Cell Signal 19: 748-760.
144. Lipnik K, Naschberger E, Gonin-Laurent N, Kodajova P, Petznek H, et al. (2010) Interferon gamma-induced human guanylate binding protein 1 inhibits mammary tumor growth in mice. Mol Med 16: 177-187.
